# First-in-human studies of seletalisib, an orally bioavailable small-molecule PI3Kδ inhibitor for the treatment of immune and inflammatory diseases

**DOI:** 10.1007/s00228-017-2205-7

**Published:** 2017-02-04

**Authors:** Eric Helmer, Mark Watling, Emma Jones, Dominique Tytgat, Mark Jones, Rodger Allen, Andrew Payne, Annelize Koch, Eugene Healy

**Affiliations:** 1 UCB Pharma, 208 Bath Road, Slough, Berkshire, SL1 3WE UK; 2 TranScrip Partners LLP, Reading, UK; 3 Veramed Limited, Twickenham, UK; 4 UCB Pharma, Braine l’Alleud, Belgium; 5 Clinical Pharmacokinetics/Pharmacometrics, Sanofi-Aventis, Deutschland GmbH, Frankfurt am Main, Germany; 6 PAREXEL, Harrow, UK; 7 Dermatopharmacology, Clinical and Experimental Sciences, Faculty of Medicine, University of Southampton, Southampton, UK

**Keywords:** Inflammatory, PI3Kδ, Phase I, Pharmacodynamic, Pharmacokinetic, Seletalisib

## Abstract

**Purpose:**

PI3Ks are potential therapeutic targets in immune-inflammatory diseases. These studies aimed to investigate the safety, tolerability and PK profile of seletalisib, a selective inhibitor of PI3Kδ in humans.

**Methods:**

These phase I, randomised, double-blind, placebo-controlled, single-centre studies (NCT02303509, NCT02207595) evaluated single and multiple oral doses of seletalisib (5–90 mg QD and 30 mg BID) in healthy adults and subjects with mild-to-moderate psoriasis (Study-1). Pharmacodynamic effects on markers of inflammation were assessed via changes in ex vivo basophil degranulation and histological assessment of psoriatic skin biopsies.

**Results:**

Seletalisib was well tolerated at doses ≤15 mg (Study-1) and ≤45 mg QD (Study-2) for 14 days. No safety concerns or dose-limiting toxicities were identified (Study-1). Incidence of gastrointestinal-related AEs was not dose related but higher incidences of rash AEs were associated with higher-dose seletalisib (Study-2 rash AEs: 18 in 12 seletalisib-treated subjects versus 1 in 1 placebo-treated subject). Mean seletalisib plasma concentration-time profiles increased with increasing doses after single and multiple dosing, with no major deviations from dose-proportionality. There was no unexpected accumulation or loss of exposure after multiple dosing (time-independent pharmacokinetic profile). Apparent *t*_1/2_ values were supportive of once-daily dosing (geometric mean t_1/2_: Study-1, 17.7–21.1 h; Study-2, 18.1–22.4 h). No clinically significant food effect was observed (Study-1). Pharmacodynamic findings demonstrated ex vivo inhibition of basophil degranulation, improvements in histological assessment of skin biopsies and other markers of psoriatic biology and preliminary evidence of target engagement in psoriatic skin tissue.

**Conclusions:**

Seletalisib safety, tolerability and pharmacokinetic/pharmacodynamic profiles support its continued clinical development in immune-inflammatory diseases.

**Electronic supplementary material:**

The online version of this article (doi:10.1007/s00228-017-2205-7) contains supplementary material, which is available to authorized users.

## Introduction

Greater understanding of the mechanisms underlying immune-inflammatory disease has driven treatment towards disease-modifying approaches, such as biological, kinase-inhibitor and cell-based therapies [1–4]. Despite these advances, suboptimal outcomes continue to be a barrier to the effective management of immune-inflammatory diseases and there remains a clinical need for novel therapeutic interventions.

PI3Ks are key enzymes regulating cell survival, proliferation and differentiation [5]. Ongoing development of small-molecule inhibitors targeting Class I PI3K isoforms (α, β, γ and δ) aims to provide enhanced therapeutic options in cancer, inflammation and autoimmunity [5]. The PI3Kα and PI3Kβ isoforms are ubiquitously expressed, whereas the PI3Kγ and PI3Kδ isoforms are expressed predominantly in leukocytes [5–8]. Pan-Class I, PI3Kα-specific and PI3Kβ-specific inhibition has been the focus of anti-tumour research, but targeted inhibition of PI3Kγ and PI3Kδ has been prioritised for the treatment of immune-inflammatory diseases [5]. PI3K isoform-specific inhibition has the potential to improve clinical responses not only where targeting multiple isoforms with a combination of isoform-specific compounds is beneficial (e.g. solid tumours, functional redundancy between PI3K isoforms) but also where it may not be (e.g. chronic lymphocytic leukaemia, autoimmune inflammatory disease) [5].

Studies in mice with a catalytically inactive knock-in of the p110δ gene (D910A) have demonstrated the pivotal role of PI3Kδ in the development and function of T, B and mast cells [9]. Pharmacologic inhibition of PI3Kδ suppresses B cell response to antigen stimulation and to cytokines, suggesting that inhibition of this pathway is likely to suppress B cell activity in autoimmune diseases [7, 10]. Furthermore, pharmacological inhibition of PI3Kδ has inhibitory effects on human T cells ex vivo [11].

Seletalisib is an orally bioavailable small-molecule with preclinical evidence supporting selective inhibition of PI3Kδ at both the biochemical and cellular level, and no apparent potential liabilities due to off-target activity (UCB data on file), thereby supporting the rationale to initiate an early clinical development program in immune and inflammatory diseases such as psoriasis, rheumatoid arthritis and primary Sjogren syndrome. This paper presents results from two clinical studies investigating the safety, tolerability and pharmacokinetic (PK) profiles of single and multiple doses of seletalisib and the pharmacodynamic (PD) effects on markers of inflammation.

Study-1 was the first-in-human study in healthy adults and subjects with mild-to-moderate psoriasis (NCT02303509), whereas Study-2 (NCT02207595) further evaluated seletalisib at higher doses in healthy adults. The primary objective of each study was to evaluate the safety and tolerability of seletalisib administered as single oral doses and as ascending multiple oral doses in healthy adults and in subjects with psoriasis (Study-1 only). A secondary objective in each study was to evaluate the PK profile of single and multiple doses of seletalisib (including preliminary assessment of the effect of food in Study-1 only) and to investigate the PD effect of seletalisib as indicated by changes in ex vivo basophil degranulation. In Study-1, exploratory objectives in one cohort of subjects with mild-to-moderate psoriasis included investigation of the effects of seletalisib on measures of cellular responses in situ (including effects on numbers of T cells, dermal dendritic cells, Langerhans cells and neutrophils) and evaluation of the effect of multiple doses of seletalisib on clinical features of plaque psoriasis.

## Methods

### Study populations

Healthy subjects (both studies) and subjects with mild-to-moderate psoriasis (Study-1 only), age 18–55 years, were enrolled by the Principal Investigators. All subjects were required to be in good physical and mental health, with body mass index (BMI) 18.0–30.0 kg/m^2^ for healthy volunteers and 18.0–35.0 kg/m^2^ for subjects with psoriasis (as the latter tend to have higher BMIs [12]), and with clinical laboratory test results, vital signs, and ECG that were within normal ranges or, if outside normal range, were isolated findings considered not clinically significant. In Study-1, subjects with psoriasis had a confirmed diagnosis of mild-to-moderate plaque-type psoriasis for ≥6 months involving ≤10% of body surface area (BSA), excluding the scalp, and to have ≥2 psoriatic lesions with ≥1 plaque at a site suitable for biopsies.

Full exclusion criteria are detailed in Online Resource S1 **.** Independent ethics committee approvals were obtained, and all subjects provided written informed consent to participate. The research ethics committee for both studies was Berkshire B Research Ethics Committee (Study 1 ref. 13/SC/0366; Study 2 ref. 14/SC/1035).

### Study designs

These were phase I, randomised, double-blind, placebo-controlled studies designed to evaluate the safety, tolerability, PK and PD profiles of seletalisib following oral administration of single ascending doses and multiple ascending doses. Study-1 was a first-in-human, double-blind (sponsor-, investigator- and subject-blind) study that had two parts: Part-A evaluated seletalisib (single ascending doses) in healthy adults (including evaluation of food effects; Table 1); Part-B evaluated seletalisib (multiple ascending doses) in healthy adults and subjects with mild-to-moderate psoriasis. Study-2 was also a double-blind study of seletalisib (single and multiple ascending doses) in healthy subjects, which investigated higher doses.

**Table 1:** Study-1 Part-A single ascending dose alternating panel study design

Sequence number	Panel 1 *N* = 17		
	Period 1	Period 2	Period 3
1 (*N* = 5)	5 mg (*n* = 5)	15 mg (*n* = 5)	5 mg (fed) (*n* = 5)
2 (*N* = 5)	Placebo (*n* = 5)	15 mg (*n* = 5)	5 mg (fed) (*n* = 5)
3 (*N* = 7^a^)	5 mg (*n* = 7)	Placebo (*n* = 4)	5 mg (fed) (*n* = 5)
	Panel 2 *N* = 18		
	Period 1	Period 2	Period 3
1 (*N* = 6^a^)	10 mg (*n* = 5)	1 mg (*n* = 5)	NA^b^
2 (*N* = 6^a^)	Placebo (*n* = 5)	1 mg (*n* = 5)	NA^b^
3 (*N* = 6^a^)	10 mg (*n* = 5)	Placebo (*n* = 5)	NA^b^

In each panel, subjects were randomised to 1 of 3 sequences and received UCB5857 or matching placebo. Panels were alternated in the following order: panel 1 period 1; panel 2 period 1; panel 1 period 2; panel 2 period 2; panel 1 period 3

*N* = total number of patients in sequence and period; *n* = number of patients receiving dose (note that patients received one or more doses); NA = not available

^a^Two subjects in sequence 3 and one subject in sequences 4, 5 and 6 discontinued; all were replaced by new subjects who received the same treatment and the same dose as those who discontinued

^b^In the absence of any emergent safety or PK data concerns, the safety review group decided to proceed to Part 2 (MAD) at a dose of 5 mg without conducting panel 2 period 3 as originally planned

Study-1 (Part-A), comprised single doses of seletalisib (1, 5, 10 and 15 mg) administered to healthy subjects using an alternating panel approach (Table 1). Panel 1 consisted of 17 subjects and panel 2 of 18 subjects. In each panel subjects were randomised to one of three sequences and received different ascending doses of seletalisib or placebo over three (panel 1) or two (panel 2) periods (Table 1). In the third period of panel 1, a preliminary assessment of food effect was made, all subjects received seletalisib 5 mg 30 min after a high-fat, high-calorie meal [13]. Preliminary findings in Study-1 (Part-A) showed no food effect, hence seletalisib and placebo were administered with food in Study-1 (Part-B) and Study-2.

In Study-1 (Part-B), a total of 27 subjects were enrolled, two cohorts of healthy subjects (*n* = 9 in each) and one cohort of subjects with mild-to-moderate psoriasis (*n* = 9). Subjects were randomised (2:1) to ascending multiple doses of seletalisib (5, 8 and 15 mg) or placebo for 14 days.

**Fig. 1:**
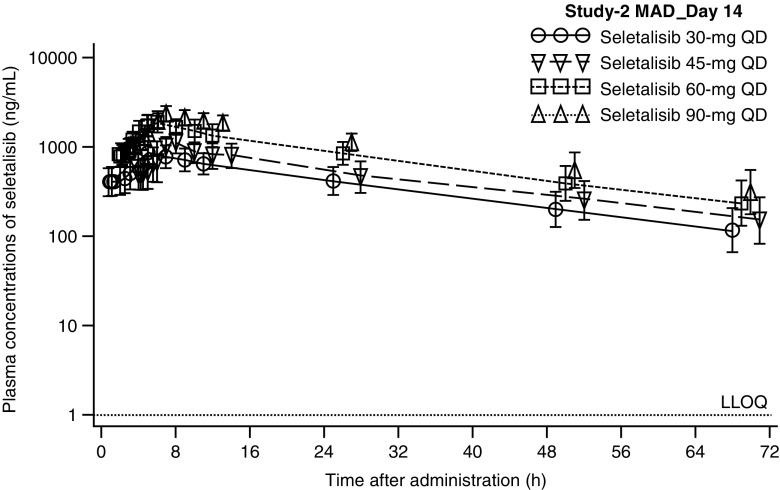
Geometric mean (95% CI) seletalisib plasma concentration-time plots (PK-PPS) Study-2 multiple dose day 14. Geometric mean and 95% CI were only calculated if at least two-thirds of the data were above the LLOQ at the respective time point. Values that were obtained after a subject had discontinued dosing were excluded. CI = confidence interval, LLOQ = lower limit of quantification, PK-PPS = pharmacokinetic per-protocol set. Data points are staggered to improve readability

In Study-2, each dose cohort had a single-dose and a multiple-dose part with administration of seletalisib or placebo on day 1 and days 1–14, respectively (Online Resource S2 Fig. 1). In total, 60 subjects were enrolled into five dose cohorts (*n* = 12 in each), and subjects were randomised (3:1) to receive seletalisib or placebo. Study-2 included initial dose cohorts of 30, 60 and 90 mg once-daily plus additional dose cohorts of 30 mg twice-daily and 45 mg once-daily.

In both studies, one sentinel subject received a single dose of seletalisib and another received a single dose of placebo before randomization of the remaining subjects in order to identify any concerning adverse events and minimise initial subject exposure at each dose level/cohort. Twenty-four-hour safety and tolerability data were reviewed in a blinded manner to assess the subjects’ safety, health and well-being prior to dosing the remaining subjects in the cohort. Dose escalation or dose reduction was determined based on review of PK and safety/tolerability data from the previously dosed cohorts.

### Pharmacokinetic assessments

Plasma concentrations of seletalisib were measured by high-performance liquid chromatography with tandem mass spectrometry (see Online Resource S1 for further details). The following PK parameters were determined in each study: AUC for seletalisib, maximum observed plasma concentration of seletalisib after a single dose (C_max_) and at steady-state (C_maxss_), time of occurrence of C_max_ (t_max_, obtained directly from the observed plasma concentration-time curves), minimum observed plasma concentration of seletalisib at steady-state immediately before the next dose would be administered (C_trough_), apparent terminal half-life, apparent volume of distribution after single dosing (Vz/F) and at steady-state (Vz_ss_/F) and apparent total body clearance after single dosing (CL/F) and at steady-state (CL_ss_/F). Accumulation factor based on AUC_(0–24)_ [R(AUC)] and accumulation factor based on C_max_ [R(C_max_)] were determined on multiple dose day 14. The following study-specific PK parameters were also determined: Study-1, total amount of seletalisib excreted in urine (Ae), renal clearance (CLr) and fraction of drug excreted in urine ; Study-2, AUC_(0–10)_ in BID dose groups, terminal elimination rate constant in plasma (λ_z_), mean residence time (MRT) and linearity factor (LF [AUC_(0–24)ss_/AUC]).

Methods for the calculation of seletalisib PK parameters are detailed in Online Resource S1.

### Pharmacodynamic assessments

The degranulation of basophils following crosslinking of FcεR1 has been shown to be PI3Kδ-dependent [14]. The activity of PI3Kδ inhibitors on basophil degranulation can be assessed by monitoring the inhibition of ex vivo IgE-mediated CD63 expression. Subjects were assessed for basophil degranulation following stimulation at multiple time points, as described in Online Resource S1.

To enable assessment of target engagement, the following exploratory PD assessments were performed at various time points prior to and following study drug administration in those subjects with mild-to-moderate psoriasis. Size and appearance of the psoriatic lesion of interest (selected at screening) were assessed by lesion severity score and lesion area. Total affected area (percentage of BSA) and Physician’s Global Assessment were assessed to evaluate overall disease severity. Histological assessments of skin biopsies (6 mm) were performed by immunofluorescent microscopy of frozen sections of lesional skin, except for haematoxylin and eosin staining and IL-17 staining which were performed on formalin fixed paraffin embedded sections. Numbers of T cells (CD3), Langerhans cells (CD1a), neutrophils (MPO) and dermal dendritic cells (CD11c) were determined.

### Safety assessments

Adverse events (AEs), serious AEs (SAEs), routine clinical laboratory parameters, vital signs and ECGs were monitored throughout the study periods and for ≤7 days post-administration of final-dose of seletalisib/placebo. The potential for seletalisib to cause gastrointestinal (GI) tract adverse effects was assessed by careful monitoring of subject reports of AEs such as abdominal pain, bloating, nausea, vomiting and alteration of bowel function (e.g. diarrhoea, constipation or bleeding). A formal safety review was conducted before each dose escalation. In Study-1 (Part-B), the potential for seletalisib to interfere with insulin signalling was assessed by monitoring blood glucose and C-peptide levels as part of the standard laboratory tests and by performance of oral glucose tolerance tests, measuring glucose and C-peptide levels, pre-dose and during exposure in all cohorts.

### Statistical analysis

In each study, the primary population for analysis was the full analysis set (FAS), which comprised all subjects who had received ≥1 dose of seletalisib or placebo. PK analysis was performed on the PK per-protocol set (PK-PPS), which was a subset of the FAS consisting of subjects with no important protocol deviations likely to affect PK variables who received seletalisib; subjects who received placebo were excluded from this analysis. In Study-1 and Study-2, sample sizes of 57 and 36 subjects, respectively, were considered sufficient to permit assessment of the safety and tolerability of seletalisib and were not based on statistical considerations of power and sample size. In Study-1 (Part-A), a panel size of 15 subjects was selected to achieve good estimates of the PK/PD parameters.

## Results

### Study disposition and demographics

**Fig. 2:**
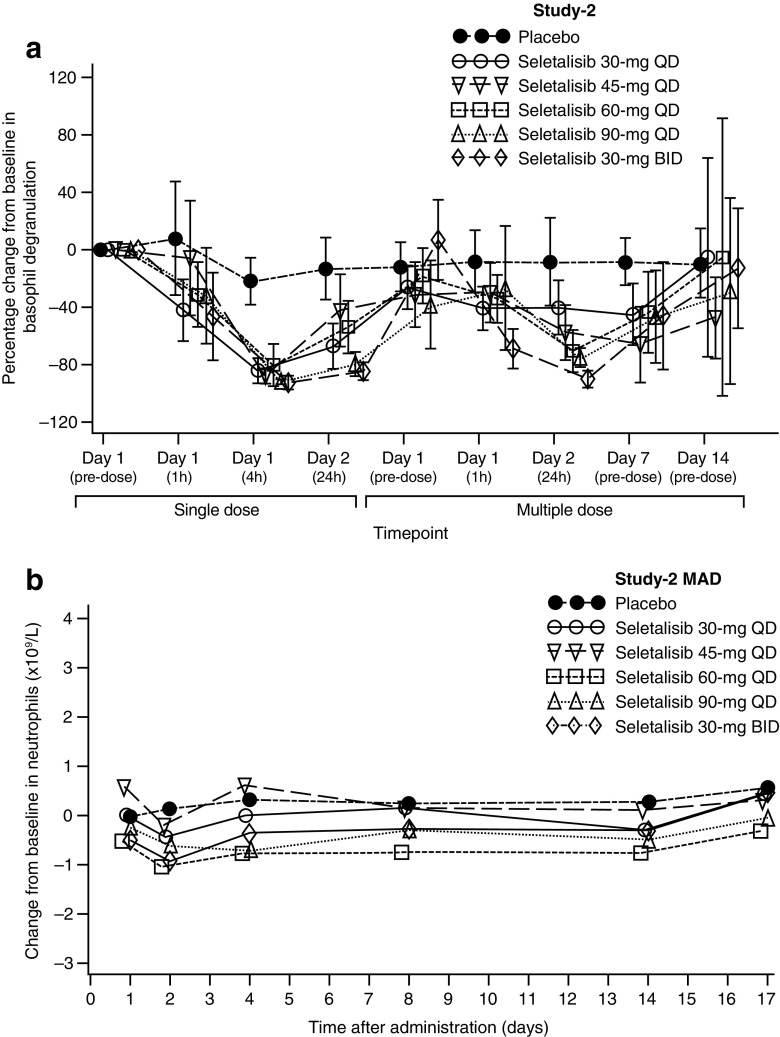
Mean percentage change from baseline in basophil degranulation (FAS) in Study-2 single and multiple doses (**a**) and mean change from baseline in neutrophils over time (FAS) Study-2 at multiple doses (**b**). Data points are staggered to improve readability

In Study-1, 248 subjects were screened for study participation, 62 of whom were randomised (Online Resource S2 Fig. 2A). The majority of screen failures were due to subject ineligibility (133 subjects [53.6%]); failed basophil degranulation tests were the most common reason for ineligibility (68 subjects [27.4%]). Overall, 35 subjects were randomised in Part-A of Study-1 and 27 were randomised in Part-B. Most subjects completed the study (30 [85.7%] in Part-A and 26 [96.3%] in Part-B); the most common reason for failing to complete the study was withdrawn consent. The first subject was enrolled on 05 August 2013, and the last subject completed the study on 25 February 2014.

In Study-2, 154 subjects were screened and 94 (61.0%) were not randomised to treatment with ‘ineligibility’ cited as the most common reason (40.3%) (Online Resource S2 Fig. 2B). Of the 60 subjects (39.0%) randomised to treatment, 52 completed the study. No placebo-treated subject discontinued the study. Overall, the most common reason for seletalisib discontinuation was ‘AE’ (*n* = 6). The highest rate of discontinuation occurred in the seletalisib 90 mg QD group (‘AE’, *n* = 2; ‘other’, *n* = 1). The first subject was enrolled on 04 August 2014, and the last subject completed the study on 28 February 2015.

In Study-1 (both parts) and Study-2, subject demographic characteristics were generally similar between treatment groups (Online Resource S2 Table 1).

### Pharmacokinetic profile

**Fig. 3:**
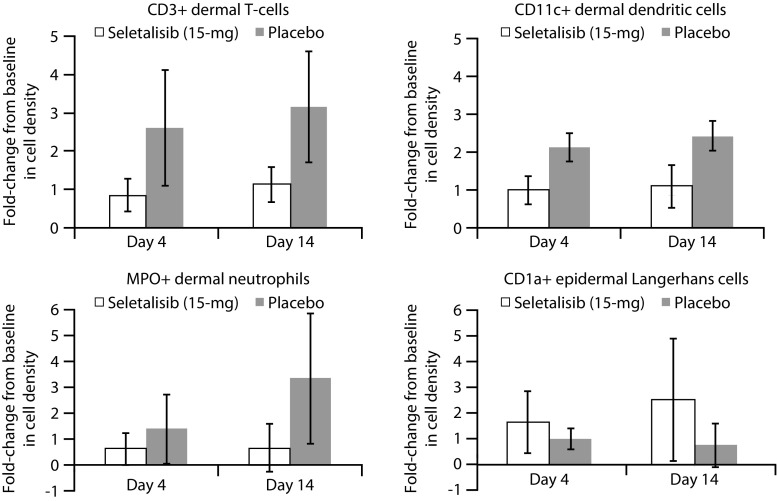
Cellular responses in lesional biopsies. Lesional biopsies were taken on days 4 and 14 from each patient, and subsequently fixed, sectioned and, using immunofluorescence, stained for CD11c (dendritic cells), CD1a (Langerhans cells), CD3 (T cells) and myeloperoxidase (MPO; neutrophils). Cells were identified using a nuclear stain. Images acquired on a slide scanner were analysed using Definiens software and, following thresholding, positive cells identified. The number of positive cells per square millimeter was calculated and the fold-change in cell density from baseline was calculated. Data are mean ± SD (placebo, *n* = 3; treated, *n* = 6)

In both studies, mean plasma concentration-time profiles increased with increasing single or multiple doses of seletalisib, and indicated a shallow biphasic disposition (Fig. 1; Online Resource S2 Fig. 3). No major deviations from dose-proportionality after single or multiple doses were observed in either study (from 1 to 90 mg; Online Resource S2 Table 2).

In Study-1 (Part-A), single-dose administration resulted in short times to peak plasma concentrations, with median t_max_ ranging from 1.8 to 3.0 h in the fasted seletalisib treatment groups (Online Resource S2 Table 3). No clinically significant food effect was observed, although median t_max_ was slightly later in the seletalisib 5 mg fed treatment group than in the seletalisib 5 mg fasted treated group (4.00 versus 2.01 h, respectively). In Study-2, single-dose seletalisib resulted in intermediate times to peak plasma concentrations, with median t_max_ ranging from 3.0 to 4.0 h (geometric mean C_max_: 517.2–1508 ng/mL) (Online Resource S2 Table 3). Moderate-to-high inter-subject variability was observed for all plasma PK parameters (GeoCV% up to 44.6%). In both studies, a moderate volume of distribution and a low clearance were observed across treatment groups. In Study-1 (Part-A), renal excretion of the parent drug was low across dose groups (geometric mean fraction of drug excreted in urine <6%). Observed *t*_½_ values were supportive of seletalisib once-daily dosing (geometric mean *t*_½_: Study-1, 17.7–21.1 h; Study-2, 18.1–22.4 h).

In each study, seletalisib multiple doses under fed conditions resulted in similar times to peak plasma concentrations (Online Resource S2 Table 4) compared to single doses. In Study-1 (Part-B), peak plasma concentrations on day 1 were achieved within 3.01–4.01 h. In Study-1 (Part-B) and Study-2, peak plasma concentrations on day 14 were achieved within 3.00–3.50 and 3.5–4.0 h, respectively. The observed accumulation ratio for both AUC R(AUC) and C_max_ R(C_max_) were broadly within the ranges of predicted accumulation ratios, based on the apparent clearance observed following single-dose administration. In Study-2, time-independent PK (LF) were observed for seletalisib 30, 45, 60 and 90 mg QD, indicating no unexpected accumulation or loss of exposure. In Study-1 (Part-A and Part-B), renal excretion of the parent drug was low across dose groups (geometric mean fraction of drug excreted in urine ≤12%).

### Pharmacodynamic profile

In both parts of Study-1, the variability of observed basophil degranulation inhibition was very high. Furthermore, only half of the seletalisib 15-mg cohort samples (Part-A) were analysed due to a technical problem. Therefore, Study-1 basophil degranulation data should be interpreted with caution. In Part-A of Study-1, inhibition of basophil degranulation was observed in all seletalisib-treated groups compared with placebo (*n* = 35) (Online Resource S2 Fig. 4A). Inhibition of basophil degranulation appeared dose-related at ≤10 mg. On day 1 of Part-B, inhibition of basophil degranulation was seen in all seletalisib groups and in the placebo group (*n* = 27), with the largest effect observed in the seletalisib 15-mg group (Online Resource S2 Fig. 4B). In Study-2, single-dose seletalisib led to 80.3–92.5% inhibition of basophil degranulation by 4 h post-dose on day 1. However, lower levels of basophil degranulation inhibition were observed after multiple-dose seletalisib on day 14 compared with day 1 (Fig. 2a).

In Study-1, the three subjects with psoriasis who received placebo showed as a group a trend towards worsening in lesion severity score, total affected area in percentage of BSA, lesion area and Physician’s Global Assessment from baseline. In the seletalisib 15-mg group, improvements in these parameters were seen in some of the six subjects with psoriasis. Due to the low number of subjects assessed and exploratory nature of this objective, no formal statistical testing was performed on the immunohistochemistry assessments. However, the descriptive summaries demonstrated some evidence of target engagement. This included a trend towards reduced numbers of dermal inflammatory cells (i.e. CD3+, CD11c+ and MPO+ cells) and a return of the resident Langerhans cells (observed as CD1a+ cells) in the six subjects with psoriasis treated with seletalisib 15 mg relative to the three placebo-treated subjects with psoriasis (Fig. 3).

### Safety/tolerability

#### Study-1

Seletalisib had an acceptable AE profile at single and multiple doses ≤15 mg daily for 14 days in healthy subjects and subjects with mild-to-moderate psoriasis (Table 2 and Online Resource S2 Table 5). There were no deaths, or discontinuations due to AEs reported in any treatment group.

**Table 2:** Adverse event profile of multiple ascending doses of seletalisib (FAS): (A) Study-1; (B) Study-2

(A) Study-1							
AE, *n* (%)	Placebo	Seletalisib 5 mg		Seletalisib 8 mg	Seletalisib 15 mg		Seletalisib Total
*N*	9	6		6	6		18
Any AE	5 (55.5)	3 (50.0)		3 (50.0)	5 (83.3)		11 (61.1)
Serious AEs	0	0		0	0		0
Discontinuations due to AEs	0	0		0	0		0
Drug-related AEs	4 (44.4)	2 (33.3)		2 (33.3)	4 (66.7)		8 (44.4)
Severe AEs	0	0		0	0		0
Deaths	0	0		0	0		0
AEs reported by >1 subject in any treatment group							
Headache	2 (22.2)	1 (16.7)		0	4 (66.7)		5 (27.8)
Back pain	0	2 (33.3)		0	0		2 (11.1)
Psoriasis	2 (22.2)	0		0	1 (16.7)		1 (5.6)
(B) Study-2							
AE, *n* (%) [#]	Placebo	Seletalisib 30 mg QD	Seletalisib 45 mg QD	Seletalisib 60 mg QD	Seletalisib 90 mg QD	Seletalisib 30 mg BID	Seletalisib total
*N*	15	9	9	9	9	9	45
Any AE	8 (53.3) [26]	5 (55.6) [9]	7 (77.8) [33]	7 (77.8) [61]	6 (66.7) [33]	7 (77.8) [33]	32 (71.1) [169]
Serious AEs	0	0	1 (11.1) [1]	1 (11.1) [1]	0	0	2 (4.4) [2]
Discontinuations due to AEs	0	0	2 (22.2) [2]	1 (11.1) [1]	2 (22.2) [2]	1 (11.1) [1]	6 (13.3) [6]
Drug-related AEs	3 (20.0) [14]	2 (22.2) [3]	4 (44.4) [21]	7 (77.8) [52]	4 (44.4) [31]	7 (77.8) [29]	24 (53.3) [136]
Severe AEs	0	0	1 (11.1) [1]	1 (11.1) [1]	1 (11.1) [1]	0	3 (6.7) [3]
Deaths	0	0	0	0	0	0	0
AEs reported by >1 subject in any treatment group							
Headache	1 (6.7)	0	1 (11.1)	5 (55.6)	2 (22.2)	3 (33.3)	11 (24.4)
Rash	1 (6.7)	0	1 (11.1)	3 (33.3)	4 (44.4)	3 (33.3)	11 (24.4)
Nausea	0	1 (11.1)	0	2 (22.2)	1 (11.1)	2 (22.2)	6 (13.3)
Oropharyngeal pain	2 (13.3)	1 (11.1)	1 (11.1)	1 (11.1)	2 (22.2)	1 (11.1)	6 (13.3)
Abdominal pain	1 (6.7)	0	0	2 (22.2)	2 (22.2)	1 (11.1)	5 (11.1)
Dizziness	0	0	1 (11.1)	2 (22.2)	1 (11.1)	1 (11.1)	5 (11.1)
Dysgeusia	1 (6.7)	0	0	3 (33.3)	2 (22.2)	0	5 (11.1)
Dermatitis, contact	0	0	3 (33.3)	0	0	1 (11.1)	4 (8.9)
Diarrhoea	1 (6.7)	0	0	0	3 (33.3)	0	3 (6.7)
Rash, generalised	0	0	1 (11.1)	2 (22.2)	0	0	3 (6.7)
Abdominal discomfort	1 (6.7)	0	0	2 (22.2)	0	0	2 (4.4)
Dry mouth	0	0	0	2 (22.2)	0	0	2 (4.4)
Furuncle	0	0	0	0	0	2 (22.2)	2 (4.4)
Hypoaesthesia oral	0	0	0	2 (22.2)	0	0	2 (4.4)
Initial insomnia	0	2 (22.2)	0	0	0	0	2 (4.4)
Regurgitation	0	0	0	2 (22.2)	0	0	2 (4.4)
Somnolence	1 (6.7)	0	0	0	0	2 (22.2)	2 (4.4)
Toothache	0	0	0	2 (22.2)	0	0	2 (4.4)

Note: In Study-1, subjects who received more than one treatment in Part-A may have been reported in multiple columns but were counted only once in the total column

AE = adverse event

^a^Although 35 subjects were randomised in Study-1 (Part-A), two subjects discontinued the study after receiving only placebo and are, therefore, not included in the seletalisib total *N* calculation

In Part-A, all AEs were of mild intensity, except for five moderate-intensity AEs: three in seletalisib-treated subjects and two in placebo-treated subjects.

In Part-B, the only AEs reported by >1 subject in any treatment group were headache, back pain and psoriasis. Psoriasis was an anticipated AE due to the inclusion of subjects with mild-to-moderate psoriasis in the 15-mg cohort. All AEs were mild in intensity, except for four moderate AEs: three in seletalisib-treated subjects and one in a placebo-treated subject.

Six AEs of potential GI disturbance were reported in subjects who received seletalisib. During Part-A, ‘abdominal distension’ was reported by one subject dosed at 5 mg. During Part-B, single events of ‘abdominal distension’, ‘diarrhoea’, ‘dyspepsia’, ‘flatulence’, and ‘nausea’ were reported by three subjects (*n* = 2 [8 mg]; *n* = 1 [15 mg]). All these GI AEs were mild, transient and resolved spontaneously within 2 days. The Part-B AEs occurred at differing times during the dosing period, with no apparent correlation to introduction or duration of dosing.

Mean values for all laboratory parameters were unremarkable, with no notable differences observed between treatment groups.

Modest reductions in neutrophil counts, assessed as not clinically significant, were seen in seletalisib-treated subjects; mean levels remained within the normal range (2.0–7.5 × 10^9^/L) in all treatment groups (Fig. 2b; Online Resource S2 Fig. 5). No clinically significant abnormalities were detected in vital signs or ECGs in any treatment group.

#### Study-2

Seletalisib had an acceptable AE profile at doses ≤45 mg QD for 14 days in healthy subjects (Table 2). No deaths were reported. SAEs were reported in two (4.4%) seletalisib-treated subjects (tonsillitis and abdominal pain). Severe AEs were reported in three seletalisib-treated subjects—abdominal pain (at 60 mg QD), headache (at 45 mg QD) and rash (at 90 mg QD); all occurred during repeated dosing, were considered treatment related, and resolved following treatment. Six subjects, all seletalisib recipients, discontinued the study due to an AE (13.3%); these AEs were all non-serious, resolved following treatment, and included maculopapular rash (*n* = 1 at 45 mg QD), pyrexia (*n* = 1 at 45 mg QD), generalised rash (*n* = 1 at 60 mg QD) and rash (*n* = 2 at 90 mg QD; *n* = 1 at 30 mg BID). The only AE of severe intensity that led to study discontinuation was a case of rash at 90 mg QD.

Thirty-two seletalisib-treated subjects (71.1%; 169 AEs) experienced an AE versus eight subjects (53.3%; 26 AEs) on placebo (Table 2); most AEs occurred during multiple dosing. The commonest seletalisib-associated AEs were headache and rash (24.4% of subjects each), which occurred more frequently than with placebo (6.7%, each). Notably, rash was observed more often at seletalisib doses ≥60 mg QD than at lower doses.

Thirty-two AEs relating to GI disturbance occurred in 10 seletalisib-treated subjects and four such AEs in two placebo-treated subjects. All but one GI AEs were of mild or moderate intensity and non-serious. The single severe SAE of ‘abdominal pain’ was reported at 60 mg QD group.

A total of 18 rash-related AEs were reported in 12 seletalisib-treated subjects and one in a placebo-treated subject (Table 2). There were no SAEs of rash and only one case was of severe intensity (at 90 mg QD). The extent and pattern of rashes were variable but appeared most frequently during the second week of dosing. In those subjects who remained on study drug, the limited time available for rashes to clear before the end of dosing precluded any meaningful conclusions regarding treatment continuity and AE resolution.

There were four infection-related AEs (two of which, ‘furuncle’ and ‘tonsillitis’, were considered treatment related) in four seletalisib-treated subjects, of mild or moderate intensity. One infection-related AE was the SAE of tonsillitis described earlier.

Laboratory, vital signs and ECG findings were generally similar to those observed in Study-1. Reductions in neutrophil counts were consistent with those observed in Study-1 (Fig. 2b, Online Resource S2 Fig. 5).

As indicated by oral glucose tolerance test results from Study-1, 10 days of dosing with seletalisib produced no significant alteration in glucose handling versus baseline.

## Discussion and conclusions

Seletalisib had acceptable AE and pharmacological profiles at single and multiple doses for 14 days (≤15 mg QD in Study-1 [maximum dose tested] and ≤45 mg QD in Study-2). There were no deaths in either study.

The absence of dose-limiting toxicities in Study-1 supported exploration of higher doses of seletalisib in Study-2. The most commonly reported AEs were headache (both studies), back pain (Study-1) and rash (Study-2). In Study-2, rash and headache were also the most commonly reported treatment-related AEs. A higher overall incidence of AEs was observed in higher-dose seletalisib groups compared with lower-dose seletalisib and placebo groups. Apart from the events of rash and headache, the majority of AEs were of mild or moderate intensity, transient and self-limiting, and showed no relationship to seletalisib dose. Modest reductions in neutrophils were detected in both studies but no dose relationship was identified, and these changes were not of clinical concern. No clinically significant abnormalities in vital signs, laboratory tests or ECGs were detected in either study.

PK results demonstrated mean plasma concentration-time profiles that increased with increasing doses of seletalisib after single and multiple dosing, and indicated a short-to-intermediate time to peak concentration and a shallow biphasic disposition. No major deviations from dose-proportionality were noted up to the maximum tested dose of seletalisib (90 mg QD). In Study-1, no food effect was observed except for the expected slight increase in time to peak plasma concentration due to delayed gastric emptying with food intake. There was no unexpected accumulation or loss of exposure indicating that the seletalisib PK profile was time independent. Only a small proportion of the total dose was recovered as unchanged drug in urine. Preclinical animal studies demonstrate that seletalisib is primarily eliminated by combined hepatic metabolism and biliary excretion [15]. It remains to be determined whether seletalisib disposition could differ in special populations (e.g. patients with hepatic impairment) or as a result of drug-drug interactions. Overall, the PK profiles from these studies are supportive of a once-daily administration.

Study-1 PD results provided preliminary evidence of target engagement in psoriatic skin tissue together with changes in other markers of psoriatic cell biology. Psoriasis is a chronic inflammatory skin disease, consisting of erythematous scaly plaques in a localised (scalp, elbows, knees) or generalised (scalp/face, trunk, limbs) distribution, resulting from immune infiltrate consisting primarily of dendritic cells (antigen presenting cells), T cells and neutrophils in affected skin. [16] Although basophils are absent from psoriatic lesions, degranulation of basophils following crosslinking of FcεR1 is dependent on PI3Kδ [14]. The ex vivo stimulation of basophil degranulation inhibition demonstrated in Study-1 suggested that PI3Kδ inhibition was achieved at all tested doses of seletalisib. However, while a drug effect was observed, high inter-subject variability was noted, which could limit the quantitative value of this assay, particularly in terms of dose selection or prediction. This appeared to align with a reduction in basophil numbers in all subjects during the transition from the UK pollen season through to autumn. There have been previous reports of atopic and non-atopic individuals demonstrating a reduction in total basophil numbers following the end of a pollen season [17]. It also appears that the basophil degranulation assay was not as sensitive as required and more susceptible to variation when only low levels of inhibitor were tested in Study 1.

Study-2 data confirmed the basophil degranulation inhibitory effects observed in Study-1; however, the magnitude of this effect appeared lower at multiple dose-day 14 than multiple dose-day 1. Notably, Study-2 basophil degranulation data variability was lower compared with Study-1 and is believed to be attributable to improved assay design via stimulation with anti-FcεR1 (instead of anti-IgE), which in-house data show is less sensitive to seasonal variation. Furthermore, the use of higher-dose seletalisib provides a clearer and less variable assay signal.

Studies of psoriasis have shown it to be a complex disease involving both Th1 and Th17 cells [18, 19], as well as a substantial role for the innate immune system [20, 21]. Some evidence of reduced dermal infiltration of immune cells, including T cells, neutrophils and dendritic cells, was observed in the seletalisib-treated group compared with the placebo group, although the effects were not statistically significant. The seletalisib group also showed some evidence of restoration of Langerhans cells in the epidermis compared with the placebo group, which is consistent with previous reports of clinical responses to other treatments in psoriasis patients [22]. A more thorough analysis of PD responses to seletalisib in the immune compartment was not undertaken due to the small number of subjects in the present study, along with the inherent variability of the required ex vivo assays. The recent identification of activating mutations in PI3Kδ associated with the primary immunodeficiency disease, activated PI3Kδ syndrome (APDS), indicates that characterisation of the effects on immunity are warranted [23].

PI3Kδ-specific inhibition has demonstrated potential in the treatment of inflammatory diseases including asthma, rheumatoid arthritis (RA) and systemic lupus erythematosus [24, 25]. Furthermore, PI3K isoform-specific inhibition is associated with a reduced toxicity risk compared with inhibition of multiple PI3K isoforms [5–8]. PI3Kδ knock-in mice, which lack functional PI3Kδ, are largely protected from imiquimod-induced dermatitis, correlating with reduced interleukin (IL)-17 in the lesion and serum [26]. PI3Kδ inhibition also inhibits IL-17 production by T cells from healthy individuals and patients with psoriasis [26]. Compared to skin from healthy individuals, high levels of expression of pAKT, a downstream marker of PI3K activity, have been reported in psoriasis indicating an active PI3K pathway in this disease [27, 28].

Patients with psoriasis were included in Study-1 (one cohort only) to enable target engagement assessment on skin biopsies. Most currently available treatments for psoriasis lead to improvements in disease severity over the course of several weeks or months; consequently, it was not expected that a 2-week exposure to seletalisib would lead to clearance of skin lesions in Study-1. Despite this, some improvements in the clinical features of plaque psoriasis were seen in some of the subjects treated with seletalisib 15 mg compared with those treated with placebo. In addition, the lower numbers of immune cells, including T cells (CD3+) and neutrophils (MPO+), in the skin biopsies from subjects who received seletalisib compared with those who received placebo suggest that seletalisib may have the potential to reduce key components of the inflammatory infiltration relevant to psoriasis. Return of the resident Langerhans cells in some seletalisib-treated subjects was also encouraging. These results are particularly promising, given that the selective PI3Kδ/γ inhibitor, duvelisib, failed to meet its phase II study primary endpoint (American College of Rheumatology 20% response rate [ACR20] at week 12) in patients with moderate-to-severe RA [29].

In conclusion, results from these studies in healthy adults and subjects with mild-to-moderate psoriasis suggest that seletalisib has an acceptable safety profile at doses of ≤45 mg QD for 14 days, with a PK profile supportive of once-daily dosing and preliminary evidence for demonstration of target engagement. Collectively, these findings support the continued clinical development of seletalisib in immune-inflammatory disorders.

## Electronic supplementary material

Online Resource 1 (DOCX 21 kb)

Online Resource 2 (DOCX 44.8 kb)

Online resource S2 Figure 1. (PDF 83 kb)

Online resource S2 Figure 2a (PDF 197 kb)

Online resource S2 Figure 2b (PDF 184 kb)

Online resource S2 Figure 3a (PDF 655 kb)

Online resource S2 Figure 3b (PDF 489 kb)

Online resource S2 Figure 3c (PDF 528 kb)

Online resource S2 Figure 3d (PDF 169 kb)

Online resource S2 Figure 4a (PDF 570 kb)

Online resource S2 Figure 4b (PDF 502 kb)

Online resource S2 Figure 5a (PDF 407 kb)

Online resource S2 Figure 5b (PDF 391 kb)

Online resource S2 Figure 5c (PDF 92 kb)
