# Supplementary material for: First-in-human studies of seletalisib, an orally bioavailable small-molecule PI3Kδ inhibitor for the treatment of immune and inflammatory diseases
Source: Eur J Clin Pharmacol. 2017 Feb 4;73(5):581–91. doi: 10.1007/s00228-017-2205-7 (PMC5384962; doi:10.1007/s00228-017-2205-7)
Supplement: Supplementary file 1 — (DOCX 21 kb) [file 228_2017_2205_MOESM1_ESM.docx]

**Online Resources S1.** Methodology

Study exclusion criteria

Subjects were not eligible for study entry if any of the following criteria were met.

*Healthy subjects and subjects with mild-to-moderate psoriasis*

- Subject was an employee or direct relative of an employee of the contract research organisation or the sponsor.
- Subject had participated in another study of an investigational medication (or a medical device) within the last 3 months or 5 x t_1/2_ of the study medication, whichever is longer, or was currently participating in another study of an investigational medication (or a medical device).
- Subject had made a blood donation (>400 mL) or had a comparable blood loss (>350 mL) within the 3 months prior to first intake of study drug.
- Subject tested positive for human immunodeficiency virus-1/2 antibody (HIV-1/2Ab), hepatitis B surface antigen or hepatitis C virus antibody.
- Subject was not willing to avoid heavy physical exertion for 2 days before drug administration and during the study.
- Subject had a history of alcohol and/or drug abuse up to 6 months before screening.
- Subject had an alcohol consumption of more than 21 units (males) or 14 units (females) of alcohol per week (1 unit of alcohol equivalent to 10 mL ethanol; for example, 330 mL of 5% alcohol by volume beer = 1.7 units; 125 mL of 12% wine = 1.5 units: 50 mL of spirits with 40% of alcohol by volume = 2 units).
- Subject tested positive for alcohol and/or drugs (urine tests) at screening or Day_-1. In Study-2, it was specified that subjects should not have consumed alcohol-containing products 72-h prior to check-in (Day_-1).
- Subject had consumed products containing caffeine- or xanthine-related substances 72-h prior to check-in (Day_-1) (Study-2 only).
- Subject had received any prescription (including hormonal replacement therapy), non-prescription medicines, including over-the-counter (OTC) remedies, herbal and dietary supplements (other than vitamins within recommended daily dose limits), or known inhibitors/inducers of CYPs 3A4, 2C9, 2C19, 2D6, 1A2, and 2A6 within 21 days or 5 x t_1/2_ of the respective drug, whichever is longer, prior to check-in (Day_-1), other than occasional use of analgesics such as paracetamol (acetaminophen), ibuprofen, or intranasal corticosteroids for seasonal rhinitis.
- Subject had consumed any grapefruit, grapefruit juice or grapefruit-containing products, Seville-style (sour) oranges, or poppy seeds, as well as nutraceuticals (St John's Wort-containing products, ginseng, kava kava, ginkgo biloba, and melatonin) within 14 days prior to check-in (Day_-1).
- Subject had a known hypersensitivity to any components of the study drug.
- Subject had current or past history of gastrointestinal ulceration.
- Subject was considered anti-IgE non-responsive (CD63 induction on basophils was <10%).
- Subject was considered anti-high-affinity IgE receptor non-responsive (CD63 induction on basophils was <10%).
- Subject had cardiovascular or cerebrovascular disease, including hypertension, angina, ischaemic heart disease, transient ischaemic attacks, stroke and peripheral arterial disease sufficient to cause symptoms and/or require therapy to maintain stable status.
- Subject had diabetes mellitus of any type requiring insulin.
- Subject had unstable/poorly controlled Type 2 diabetes mellitus, defined as glycosylated haemoglobin type A1c (HbA1c) level ≥8.5% or glucose intolerant (Study-1 only).
- Subject had an active infection (e.g. sepsis, pneumonia, abscess), a history of latent, chronic or recurrent infections (e.g. tuberculosis, recurrent sinusitis, genital herpes, urinary tract infections) or was at risk of infection (surgery, trauma, infection requiring antibiotics, history of skin abscesses) within 3 months prior to study drug administration, or experienced a significant episode of gastroenteritis (defined as loose stools associated with abdominal pain and/or fever) during the 7 days prior to study drug administration.
- Subject had a history of positive tuberculosis (TB) test or evidence of possible TB or latent TB infection at screening (Study-1, interferon gamma release assay [IGRA] testing; Study-2, QuantiFERON^®^ Gold Test).
- Subject had received live attenuated vaccination within 3 months or any other type of vaccine within 4 weeks prior to screening or intended to have such a vaccination during the study period.
- Subject had any of the following haematology values at screening:
- Haemoglobin <11 g/dL for women and <13 g/dL for men
- Absolute neutrophil count <1.5 x 10^9^/L.
- Subject had an abnormality in the 12-lead ECG that, in the opinion of the investigator, increased the risks associated with participating in the study. In addition, any subject with any of the following findings was excluded:
- QTcF interval >450 ms in ≥2 of 3 ECGs
- Bundle branch blocks and other conduction abnormalities (other than mild first-degree atrioventricular block) such that the PR interval was ≥220 ms
- Irregular rhythms other than sinus arrhythmia or occasional, rare supraventricular or rare ventricular ectopic beats
- T-wave configurations not of sufficient quality for assessing QT interval duration.
- Subject had active neoplastic disease or history of neoplastic disease within 5 years of screening (except for basal or squamous cell carcinoma of the skin or carcinoma *in situ* that had been definitively treated with standard of care) (Study-1 only).
- Subject had any other acute or chronic illness that could have posed a threat or harm to the subject.
- Subject was legally institutionalized or had a mental health condition or related care provision (e.g. guardianship) that would have impeded the subject from providing voluntary informed consent to participate in the study.

*Healthy subjects only*

- Subject had used nicotine-containing products (including, but not limited to, cigarettes, pipes, cigars, chewing tobacco, nicotine patch or nicotine gum) within 6 weeks prior to check-in (Day_-1) or anticipated an inability to abstain from these products for the duration of the study.
- Subject had a high consumption of xanthine-containing products (≥300 mg (Study-1) or (≥500 mg (Study-2) of xanthine equivalent per day [1 cup of coffee ≈ 100 mg of caffeine; 1 cup of tea ≈ 30 mg of caffeine; 1 glass of cola ≈ 20 mg of caffeine]).

*Subjects with mild-to-moderate psoriasis only*

- Subject had received systemic non-biologic psoriasis therapy (methotrexate, steroids, cyclophosphamide) or psoralen plus ultraviolet A/ultraviolet A phototherapy within 4 weeks prior to screening.
- Subject had received treatment with biologic agents within 12 months prior to the study.

**Seletalisib assay**

Plasma concentrations of seletalisib were measured by a validated analytical method. After addition of a tetra-deuterated internal standard to a 50 µL human plasma sample, seletalisib was extracted from plasma by solid phase extraction (SPE) using 30mg cartridges in the 96 well-plate format. Samples were loaded on to the SPE plate, washed and eluted. The resulting extracts were evaporated to dryness and then reconstituted in small volume of mobile phase ready for injection. The target compound and internal standard were separated by reverse phase high performance liquid chromatography (HPLC) with gradient elution using a Waters BEH C18 50 × 2.1 mm, 1.7 μm analytical column, Waters Acquity Sample Manager and Binary Solvent Manager HPLC system. Detection was performed by electrospray mass spectrometry (ESI/MS/MS) with an Applied Biosystems API5000 mass spectrometer using multiple reaction monitoring (MRM) in positive ionization mode. MRM transitions of 483.2 m/z > 395.1 m/z and 489.2 > 401.1 m/z were monitored to measure seletalisib and the internal standard, respectively. The internal standard MRM transition monitored the D4 and 37Cl isotope-containing precursor and product ions. The quantitative determination of seletalisib was validated over the range 1.00 to 1000 ng/mL. Overall inter-run precision and accuracy at concentrations of 3.00, 30.0 and 800 ng/mL was ≤5.7% and ≤10.0%, respectively.

**Seletalisib pharmacokinetic assessments**

Seletalisib PK parameters were calculated via non-compartmental analysis methods from the individual concentration-time data using Phoenix WinNonlin version 6.2 (Certara, Princeton, NJ, USA). Urine (Study-1 only) and plasma PK parameters of seletalisib were reported for each subject and summarised by study part and dose group. Geometric mean and percentage geometric coefficient of variation (GeoCV%) were reported for all parameters except for t_max_, which was reported using median and range. Dose proportionality for AUC and C_max_ at Day_1 (Study-1 [Part-A & Part-B]; Study-2, single ascending dose-Day_1) and at steady state at Day_14 (Study-1 [Part-B]; Study-2, multiple ascending dose-Day_14) was examined via the power model and reported graphically. In Study-1, an assessment of food effect (fed versus fasted) was made for C_max_ and AUC from the data for subjects who received the same dose in fed and fasted states in Part-A.

**Assessment of basophil degranulation**

Degranulation of basophils was determined in whole blood by monitoring increased cell surface CD63 expression utilizing flow cytometry in an *ex vivo* stimulation assay driven by anti-IgE (Study-1) or anti-FcεR1 (Study-2). Briefly, blood was collected in lithium-heparin tubes (BD Biosciences, Oxford, UK) and stimulated with IgE or FcεRI cross-linking antibodies (eBiosciences, Hatfield, UK) for 12 min at 37°C. Following red blood cell lysis by addition of BD FACSlyse (BD Biosciences, Oxford, UK), samples were centrifuged and the supernatant discarded. The cell pellet was washed in PBS and re-suspended in fixative (5% formaldehyde in PBS). CD63 expression was determined on basophils (side-scatter low, CD123+HLA-DR-) and detected by flow cytometry using the BD antibody cocktail containing CD63, CD123, and anti-HLA-DR.
