# Supplementary material for: First-in-human studies of seletalisib, an orally bioavailable small-molecule PI3Kδ inhibitor for the treatment of immune and inflammatory diseases
Source: Eur J Clin Pharmacol. 2017 Feb 4;73(5):581–91. doi: 10.1007/s00228-017-2205-7 (PMC5384962; doi:10.1007/s00228-017-2205-7)
Supplement: Supplementary file 2 — (DOCX 44.8 kb) [file 228_2017_2205_MOESM2_ESM.docx]

# Online Resources S2.

**S2 Table 1.** Subject demographics (FAS): (A) Study-1; (B) Study-2

| **(A) Study-1** | | | | | | | | | | | |
| --- | --- | --- | --- | --- | --- | --- | --- | --- | --- | --- | --- |
|  | **Part-A (single ascending doses)** | | | | | **Part-B (multiple ascending doses)** | | | | | |
| **Characteristic** | **Panel 1** | **Panel 2** | | **Total** | | **Placebo** | | **Seletalisib 5 mg** | **Seletalisib 8 mg** | **Seletalisib 15 mg** | **Total** |
| ***N*** | 17 | 18 | | 35 | | 9 | | 6 | 6 | 6 | 27 |
| **Age, years  Mean (SD)  Range** | 35.0 (9.4) 21–54 | 33.3 (9.3) 22–52 | | 34.1 (9.2) 21–54 | | 35.6 (7.0) 24–44 | | 37.3 (12.8) 23–52 | 34.0 (5.0) 29–43 | 37.5 (10.4) 27–55 | 36.0 (8.6) 23–55 |
| **Male, *n* (%)** | 17 (100) | 18 (100) | | 35 (100) | | 9 (100) | | 6 (100) | 6 (100) | 6 (100) | 27 (100) |
| **BMI, kg/m^2^  Mean (SD)  Range** | 24.7 (2.1) 21.2–28.9 | 24.7 (2.2) 21.0–28.0 | | 24.7 (2.1) 21.0–28.9 | | 25.5 (3.2) 21.1–29.6 | | 24.1 (1.8) 22.3–26.5 | 24.3 (3.0) 20.2–28.9 | 26.4 (2.8) 23.6–31.3 | 25.1 (2.8) 20.2–31.3 |
| **BSA, m^2^  Mean (SD)  Range** | 1.95 (0.12) 1.68–2.16 | 1.95 (0.17) 1.76–2.34 | | 1.95 (0.15) 1.68–2.34 | | 1.99 (0.17) 1.76–2.25 | | 1.97 (0.10) 1.81–2.09 | 1.88 (0.12) 1.75–2.04 | 2.12 (0.17) 1.91–2.33 | 1.99 (0.16) 1.75–2.33 |
| **Racial group, *n* (%)  Asian  Black or African American  Caucasian/Black African  Black/Asian  White/Algerian  White/Black  White** | 1 (5.9) 1(5.9) 1 (5.9) 0 0 0 14 (82.4) | 3 (16.7) 1 (5.6) 0 0 0 0 14 (77.8) | | 4 (11.4) 2 (5.7) 1 (2.9) 0 0 0 28 (80.0) | | 1 (11.1) 0 0 1 (11.1) 1 (11.1) 0 6 (66.7) | | 0 0 0 0 0 0 6 (100) | 0 0 0 0 0 1 (16.7) 5 (83.3) | 0 0 0 0 0 0 6 (100) | 1 (3.7) 0 0 1 (3.7) 1 (3.7) 1 (3.7) 23 (85.2) |
| **Ethnicity, *n* (%)  Hispanic or Latino  Not Hispanic or Latino** | 1 (5.9) 16 (94.1) | 0 18 (100) | | 1 (2.9) 34 (97.1) | | 0 9 (100) | | 0 6 (100) | 0 6 (100) | 0 6 (100) | 0 27 (100) |
| **(B) Study-2** | | | | | | | | | | | |
| **Characteristic** | **Placebo** | | **Seletalisib 30 mg QD** | | **Seletalisib 45 mg QD** | | **Seletalisib 60 mg QD** | | **Seletalisib 90 mg QD** | **Seletalisib 30 mg BID** | **Total** |
| ***N*** | 15 | | 9 | | 9 | | 9 | | 9 | 9 | 60 |
| **Age, years  Mean (SD)  Range** | 36.6 (10.7) 21–54 | | 37.3 (9.4) 24–51 | | 40.4 (10.2) 23–55 | | 30.3 (4.3) 25–39 | | 38.1 (10.7) 22–54 | 35.1 (7.2) 21–45 | 36.4 (9.4) 21–55 |
| **Male, *n* (%)** | 15 (100) | | 9 (100) | | 9 (100) | | 9 (100) | | 9 (100) | 9 (100) | 60 (100) |
| **BMI, kg/m^2^  Mean (SD)  Range** | 25.4 (2.8) 21.0–29.9 | | 25.1 (1.7) 21.5–26.8 | | 26.1 (2.0) 22.8–28.2 | | 24.0 (2.5) 21.7–29.0 | | 25.3 (2.3) 21.7–29.0 | 23.9 (3.2) 19.6–28.6 | 25.0 (2.5) 19.6–29.9 |
| **Racial group, *n* (%)  Asian  Black or African American  White  Other/mixed** | 0 1 (6.7) 12 (8.0) 2 (13.3) | | 1 (11.1) 2 (22.2) 6 (66.7) 0 | | 1 (11.1) 1 (11.1) 7 (77.8) 0 | | 1 (11.1) 0 7 (77.8) 1 (11.1) | | 0 2 (22.2) 7 (77.8) 0 | 0 1 (11.1) 7 (77.8) 1 (11.1) | 3 (5.0) 7 (11.7) 46 (76.7) 58 (96.7) |
| **Ethnicity, *n* (%)  Hispanic or Latino  Not Hispanic or Latino** | 2 (13.3) 13 (86.7) | | 0 9 (100) | | 0 9 (100) | | 0 9 (100) | | 0 9 (100) | 0 9 (100) | 2 (3.3) 58 (96.7) |

BID=twice daily; BMI=Body mass index; BSA=Body surface area; SD=Standard deviation; QD=Once daily.

**S2 Table 2.** Dose proportionality of PK parameters: (A) Study-1; (B) Study 2

| **(A) Study-1** | | | | | |
| --- | --- | --- | --- | --- | --- |
| **Parameter** | **Effect** | **Estimate** | **Estimated SE** | **95% CI** | **CV^a^ (%)** |
| **Part A** |  |  |  |  |  |
| **AUC (h*ng/mL)** | Intercept | 6.19 | 0.06 |  | 7.9 |
|  | Dose (beta) | 1.02 | 0.02 | 0.97, 1.07 |  |
| **AUC_(0-t)_ (h*ng/mL)** | Intercept | 6.10 | 0.06 |  | 8.5 |
|  | Dose (beta) | 1.01 | 0.02 | 0.97, 1.06 |  |
| **C_max_ (ng/mL)** | Intercept | 3.25 | 0.06 |  | 14.6 |
|  | Dose (beta) | 0.97 | 0.03 | 0.97, 1.04 |  |
| **Part B Day 1** |  |  |  |  |  |
| **AUC_(0-t)_ (h*ng/mL)** | Intercept | 6.04 | 0.32 |  | 28.2 |
|  | Dose (beta) | 0.83 | 0.14 | 0.53, 1.14 |  |
| **C_max_ (ng/mL)** | Intercept | 3.43 | 0.26 |  | 23.3 |
|  | Dose (beta) | 0.87 | 0.12 | 0.62, 1.13 |  |
| **Part B Day 14** |  |  |  |  |  |
| **C_maxss_ (ng/mL)** | Intercept | 3.95 | 0.37 |  | 32.1 |
|  | Dose (beta) | 0.78 | 0.17 | 0.41, 1.15 |  |
| **AUC_(0-24)_ (h*ng/mL)** | Intercept | 6.81 | 0.46 |  | 40.4 |
|  | Dose (beta) | 0.69 | 0.21 | 0.23, 1.15 |  |
| **(B) Study-2** | | | | | |
| **Parameter** | **Effect** | **Estimate** | **Estimated SE** | **95% CI** | **CV (%)** |
| **Single-dose Day 1** |  |  |  |  |  |
| **AUC (h*ng/mL)** | Intercept | 5.75 | 0.52 |  | 32.2 |
|  | Dose (beta) | 1.06 | 0.13 | 0.80, 1.33 |  |
| **C_max_ (ng/mL)** | Intercept | 2.69 | 0.47 |  | 29.2 |
|  | Dose (beta) | 1.04 | 0.12 | 0.80, 1.29 |  |
| **Multiple-dose Day 14** |  |  |  |  |  |
| **C_maxss_ (ng/mL)** | Intercept | 3.52 | 0.55 | 0.67, 1.24 | 30.3 |
|  | Dose (beta) | 0.96 | 0.14 |  |  |
| **AUC_(0-24)_ (h*ng/mL)** | Intercept | 6.14 | 0.62 | 0.67, 1.31 | 34.2 |
|  | Dose (beta) | 0.99 | 0.16 |  |  |

Note: in Study-1 Part A, only doses given in the fasted state were included in the analysis; in Study-2, only treatment groups dosed once daily were included in the analysis.

AUC=Area under plasma concentration-time curve from zero to infinity; C_max_=Maximum plasma concentration; C_maxss_= Minimum drug concentration at steady-state.

^a^Intra-individual CV.

**S2 Table 3.** Pharmacokinetic parameters of single ascending doses of seletalisib (PK-PPS): (**A**) Study-1 (plasma and urine); (**B**) Study-2 (plasma)

| **(A) Study-1** | | | | | |
| --- | --- | --- | --- | --- | --- |
| **Parameter^a^** | **Seletalisib 1 mg** | **Seletalisib 5 mg** | **Seletalisib 5 mg fed** | **Seletalisib 10 mg** | **Seletalisib 15 mg** |
| ***N*** | 10 | 12 | 15 | 10 | 10 |
| **AUC, h*ng/mL** | 518 (33.4) | 2287 (34.3) | 2680 (44.6) | 5174 (17.3) | 7759 (25.4) |
| **AUC_(0–t)_, h*ng/mL** | 473 (33.4) | 2097 (33.1) | 2480 (43.3) | 4798 (15.3) | 7005 (23.9) |
| **C_max_, ng/mL** | 26.7 (27.0) | 109 (25.1) | 105 (27.2) | 279 (16.1) | 313 (17.6) |
| **t_max_, h** | 1.77 (1.50–4.02) | 2.01 (1.50–4.00) | 4.00 (1.98–9.98) | 1.77 (0.98–4.00) | 3.00 (1.98–4.02) |
| **t_1/2_, h** | 18.5 (25.2) | 18.8 (30.8) | 17.7 (21.7) | 19.4 (22.3) | 21.1 (15.0) |
| **Vz/F, L** | 51.5 (22.9) | 59.2 (31.2) | 47.6 (35.0) | 54.0 (19.0) | 58.7 (24.6) |
| **CL/F, L/h** | 1.93 (33.4) | 2.19 (34.3) | 1.87 (44.6) | 1.93 (17.3) | 1.93 (25.4) |
| **Ae_(0–t)_, mg** | 0.058 (56.6) | 0.269 (35.3) | 0.241 (49.6) | 0.476 (66.0) | 0.689 (33.6) |
| **CL_r_, L/h** | 0.112 (76.3) | 0.117 (37.9) | 0.090 (73.2) | 0.092 (67.5) | 0.089 (55.3) |
| **Fe,%** | 5.79 (56.6) | 5.37 (35.3) | 4.81 (49.6) | 4.76 (66.0) | 4.59 (33.6) |
| **(B) Study-2** | | | | | |
| **Parameter^a^** | **Seletalisib 30 mg QD** | **Seletalisib 45 mg QD** | **Seletalisib 60 mg QD** | **Seletalisib 90 mg QD** | **Seletalisib 30 mg BID** |
| ***N*** | 9 | 9 | 9 | 9 | 9 |
| **AUC_(0–10)_, h*ng/mL** | — | — | — | — | 3498 (15.4) |
| **AUC_(0–t)_, h*ng/mL** | 10244 (27.8) | 15134 (31.4) | 25109 (30.7) | 31183 (31.7) | — |
| **AUC_(0–24)_, h*ng/mL** | 6489 (24.3) | 9284 (26.0) | 16300 (28.9) | 19659 (30.3) | — |
| **AUC, h*ng/mL** | 11332 (30.4) | 17252 (33.0) | 27373 (32.3) | 34876 (33.3) | — |
| **C_max_, ng/mL** | 517.2 (24.5) | 690.8 (23.6) | 1248 (29.5) | 1508 (32.2) | — |
| **t_max_, h** | 4.0 (3.0–4.0) | 4.0 (2.0–6.0) | 3.0 (3.0–4.1) | 4.0 (3.0–4.1) | — |
| **MRT, h** | 22.1 (8.7) | 23.0 (10.2) | 21.5 (7.2) | 22.4 (7.7) | — |
| **t_1/2_, h** | 19.5 (14.0) | 22.4 (19.8) | 18.1 (17.0) | 20.8 (19.2) | — |
| **Vz/F, L** | 74.4 (24.7) | 84.2 (35.1) | 57.2 (27.5) | 77.3 (29.4) | — |
| **CL/F, L/h** | 2.65 (30.4) | 2.61 (33.0) | 2.19 (32.3) | 2.58 (33.3) | — |

^a^All values geometric mean (% geometric coefficient of variation), except for t_max_ (median and range).

Ae_(0-t)_=total amount of seletalisib excreted in urine from time 0 to time t; AUC=area under the plasma concentration-time curve from time 0 to infinity; AUC_(0-t)_=area under the plasma concentration-time curve from time 0 to last quantifiable concentration; BID=twice daily; CL/F=apparent total body clearance after single dosing; CLr=renal clearance; C_max_=maximum observed plasma concentration of seletalisib after a single dose; Fe=fraction of drug excreted into urine in %; MRT=mean residence time; t_1/2_=apparent terminal half-life; tmax=time of occurrence of C_max_; VZ/F=apparent volume of distribution after single dosing.

**S2 Table 4.** Pharmacokinetic parameters of multiple ascending doses of seletalisib (PK-PPS): (**A**) Study-1 on Days 1 and 14 (plasma and urine); (**B**) Study-2 on Day_14 (plasma)

| **(A) Study-1** | | | | | | | |
| --- | --- | --- | --- | --- | --- | --- | --- |
| **Parameter^a^** | **Seletalisib 5 mg** | | **Seletalisib 8 mg** | | | **Seletalisib 15 mg** | |
| ***N*** | 6 | | 6 | | | 6 | |
| **Day_1** |  | |  | | |  | |
| **AUC_(0–24)_, h*ng/mL** | 1557 (20.1) | | 2607 (43.7) | | | 3942 (14.5) | |
| **C_max_, ng/mL** | 122 (13.5) | | 201 (38.4) | | | 322 (8.8) | |
| **t_max_, h** | 4.01 (2.98–4.03) | | 4.00 (2.00–5.93) | | | 3.01 (2.98–4.00) | |
| **Ae_(0–24)_, mg** | 0.201 (32.2) | | 0.340 (51.9) | | | 0.812 (48.2) | |
| **CL_r_, L/h** | 0.129 (26.3) | | 0.130 (76.7) | | | 0.206 (54.9) | |
| **Fe_(0–24)_,%** | 4.02 (32.2) | | 4.24 (51.9) | | | 5.42 (48.2) | |
| **Day_14** |  | |  | | |  | |
| **AUC_(0–24)_, h*ng/mL** | 2537 (36.2) | | 4366 (50.2) | | | 5463 (29.9) | |
| **C_maxss_, ng/mL** | 175 (29.1) | | 285 (41.7) | | | 414 (23.0) | |
| **t_max_, h** | 3.00 (1.50–4.00) | | 3.50 (2.98–4.02) | | | 3.00 (1.50–4.00) | |
| **t_1/2_, h** | 22.0 (22.6) | | 22.8 (19.2) | | | 22.5 (36.0) | |
| **C_trough_, ng/mL** | 66.5 (47.3) | | 113 (67.8) | | | 140 (35.0) | |
| **Vz_ss_/F, L** | 62.5 (20.0) | | 60.3 (40.1) | | | 89.0 (24.9) | |
| **CL_ss_/F, L/h** | 1.97 (36.2) | | 1.83 (50.2) | | | 2.75 (29.9) | |
| **R(AUC)** | 1.63 (22.8) | | 1.68 (17.9) | | | 1.44 (22.6) | |
| **R(C_max_)** | 1.43 (30.0) | | 1.42 (18.3) | | | 1.32 (16.6) | |
| **Ae_(0–24)_, mg** | 0.340 (40.0) | | 0.608 (26.3) | | | 1.80 (52.8) | |
| **CL_r_, L/h** | 0.134 (45.5) | | 0.139 (55.8) | | | 0.329 (70.7) | |
| **Fe_(0–24)_,%** | 6.80 (40.0) | | 7.60 (26.3) | | | 12.0 (52.8) | |
| **(B) Study-2** | | | | | | | |
| **Parameter^a^** | **Seletalisib 30 mg QD** | **Seletalisib 45 mg QD** | | **Seletalisib 60 mg QD** | **Seletalisib 90 mg QD** | | **Seletalisib 30 mg BID** |
| ***N*^b^** | 8 | 7 | | 8 | 6 | | 8 |
| **Day_14** |  |  | |  |  | |  |
| **AUC_(0–10)_, h*ng/mL** | — | — | | — | — | | 10565 (16.9) |
| **AUC_(0–t)_, h*ng/mL** | 24878 (46.5) | 30104 (44.5) | | 51639 (39.1) | 67643 (25.6) | | — |
| **AUC_(0–24)_, h*ng/mL** | 14087 (39.1) | 16963 (36.9) | | 30048 (32.5) | 38636 (19.7) | | — |
| **C_maxss_, ng/mL** | 897.8 (35.4) | 1114 (27.7) | | 1957 (30.2) | 2355 (17.8) | | — |
| **t_max_, h** | 4.0 (3.0–4.0) | 4.0 (3.0–4.0) | | 3.5 (2.0–6.0) | 4.0 (4.0–4.0) | | — |
| **MRT, h** | 24.9 (9.0) | 25.1 (8.9) | | 24.2 (10.2) | 24.7 (9.5) | | — |
| **t_1/2_, h** | 24.6 (21.1) | 28.5 (34.8) | | 24.6 (26.2) | 24.6 (23.7) | | — |
| **Vz_ss_/F, L** | 64.8 (24.2) | 74.5 (27.9) | | 56.7 (33.3) | 68.0 (23.9) | | — |
| **CL_ss_/F, L/h** | 2.13 (39.1) | 2.65 (36.9) | | 2.00 (32.5) | 2.33 (19.7) | | — |
| **R(AUC)** | 2.214 (21.6) | 1.912 (33.6) | | 1.961 (31.6) | 2.036 (27.7) | | — |
| **R(C_max)_** | 1.771 (23.1) | 1.704 (33.9) | | 1.684 (35.5) | 1.681 (26.3) | | — |
| **LF** | 1.275 (16.7) | 1.028 (26.4) | | 1.160 (31.8) | 1.127 (25.9) | | — |

^a^All values geometric mean (% geometric coefficient of variation), except for t_max_ (median and range) and LF (no units).
^b^Nine subjects in each dose cohort (PK parameters only calculated if at least two-thirds of the parameters were determined properly [i.e., non-calculated and non-flagged]).

Ae_(0-24)_=total amount of seletalisib excreted in urine from time 0 to time 24 hours; AUC=area under the plasma concentration-time curve from time 0 to infinity; AUC_(0-t)_=area under the plasma concentration-time curve from time 0 to last quantifiable concentration; BID=twice daily; CL/F=apparent total body clearance after single dosing; CLr=renal clearance; C_max_=maximum observed plasma concentration of seletalisib after a single dose; C_max, ss_=maximum observed plasma concentration of seletalisib at staedy state; c_trough_=minimum observed plasma concentration of seletalisib at steady-state immediately before the next dose would be administered; Fe_(0-24)_=fraction of drug excreted into urine from time 0 to 24 hours; LF=linearity factor; MRT=mean residence time; R(AUC)=accumulation factor based on AUC(0–24); R(C_max_)=accumulation factor based on C_max_; t_1/2_=apparent terminal half-life; t_max_=time of occurrence of C_max_; VZ_ss_/F=apparent volume of distribution at steady state

**S2 Table 5.** Safety and tolerability of single ascending doses of seletalisib (FAS): (Study-1)

| **AE, *n* (%)** | | **Placebo** | **Seletalisib 1 mg** | **Seletalisib 5 mg** | **Seletalisib 5 mg fed** | **Seletalisib 10 mg** | **Seletalisib 15 mg** | **Seletalisib Total** |
| --- | --- | --- | --- | --- | --- | --- | --- | --- |
| ***N*** |  | 19 | 10 | 12 | 15 | 10 | 10 | 33^a^ |
| **Any AE** |  | 5 (26.3) | 1 (10.0) | 5 (41.7) | 2 (13.3) | 3 (30.0) | 3 (30.0) | 13 (39.4) |
| **Serious AEs** |  | 0 | 0 | 0 | 0 | 0 | 0 | 0 |
| **Discontinuations due to AEs** |  | 0 | 0 | 0 | 0 | 0 | 0 | 0 |
| **Drug-related AEs** |  | 1 (5.3) | 0 | 1 (8.3) | 0 | 1 (10.0) | 0 | 2 (6.1) |
| **Severe AEs** |  | 0 | 0 | 0 | 0 | 0 | 0 | 0 |
| **Deaths** |  | 0 | 0 | 0 | 0 | 0 | 0 | 0 |
| **AEs reported by >1 subject in any treatment group  Headache** | | 0 | 0 | 2 (16.7) | 1 (6.7) | 1 (10.0) | 1 (10.0) | 4 (12.1) |

Note: Subjects who received more than one treatment in Part-A may have been reported in multiple columns but were counted only once in the total column.

^a^Although 35 subjects were randomised in Part-A, two subjects discontinued the study after receiving only placebo and are therefore not included in the sletalisib total *N* calculation.

AE=adverse event; FAS=full analysis set

# Figures

**S2 Fig. 1**

Study design: Study-2

F/U, follow-up; MD, multiple-dose; SD, single-dose; SRG, Safety Review Group.
Doses tested: Cohort-1, 30 mg QD; Cohort-2, 60 mg QD; Cohort-3, 90 mg QD; Cohort-4, 30 mg BID; Cohort-5: 45 mg QD.
Twelve subjects in each cohort (seletalisib, *n* = 9; placebo, *n* = 3).

**S2 Fig. 2**

Subject disposition: (A) Study-1; (B) Study-2

FAS, full analysis set; PD-PPS, pharmacodynamic per-protocol set; PK-PPS, pharmacokinetic per-protocol set.

*One subject failed the drugs of abuse test on readmission for the second study period, and one subject was unable to attend some study visits.

**Subjects who were only administered placebo were excluded from the PK-PPS.

**S2 Fig. 3**

Geometric mean (95% CI) seletalisib plasma concentration-time plots (PK-PPS): Study-1 (A) single dose, (B) multiple dose Day_1, (C) multiple dose Day_14; Study-2 (D) single dose

BLQ, below the limit of quantification; CI, confidence interval; LLOQ, lower limit of quantification; PK-PPS, pharmacokinetic per-protocol set.
Geometric mean and 95% CI were only calculated if at least two-thirds of the data were above the LLOQ at the respective time point.
Figs. 1D: values that were obtained after a subject had discontinued dosing were excluded. For treatment groups 30 mg QD, 45 mg QD, 60 mg QD and 90 mg QD at time points 0.25, 0.5, and 1 hour, the BLQ values were replaced by LLOQ/2.
Data points in Figs. 1D are staggered to improve readability.

**S2 Fig. 4**

Mean percentage change from baseline in basophil degranulation (FAS): Study-1 (A) single dose, (B) multiple dose

**S2 Fig. 5**

Mean change from baseline in neutrophils over time (FAS) in Study-1 (A) single dose, (B) multiple dose, Study-2 (C) single dose

Data points in Fig. 3C are staggered to improve readability.
