# Supplementary figures and images for: First-in-human studies of seletalisib, an orally bioavailable small-molecule PI3Kδ inhibitor for the treatment of immune and inflammatory diseases
Source: Eur J Clin Pharmacol. 2017 Feb 4;73(5):581–91. doi: 10.1007/s00228-017-2205-7 (PMC5384962; doi:10.1007/s00228-017-2205-7)

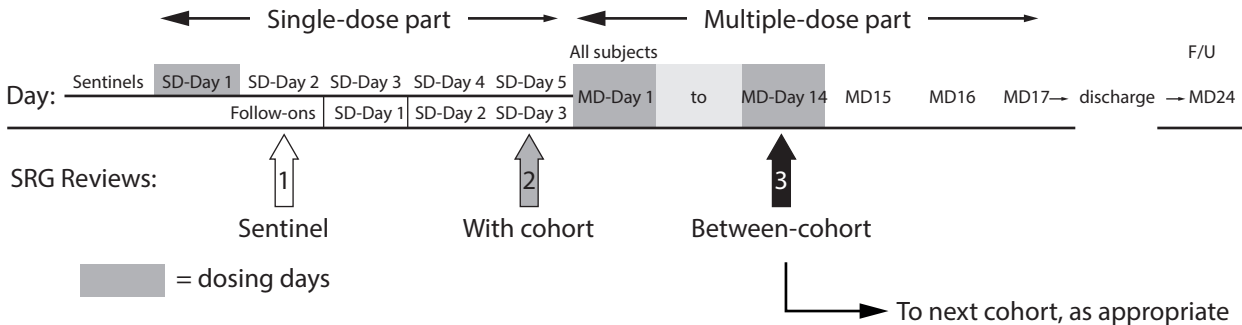

Supplement: Supplementary file 3 — Figure 1. (PDF 83 kb) [file 228_2017_2205_MOESM3_ESM.pdf]

**A**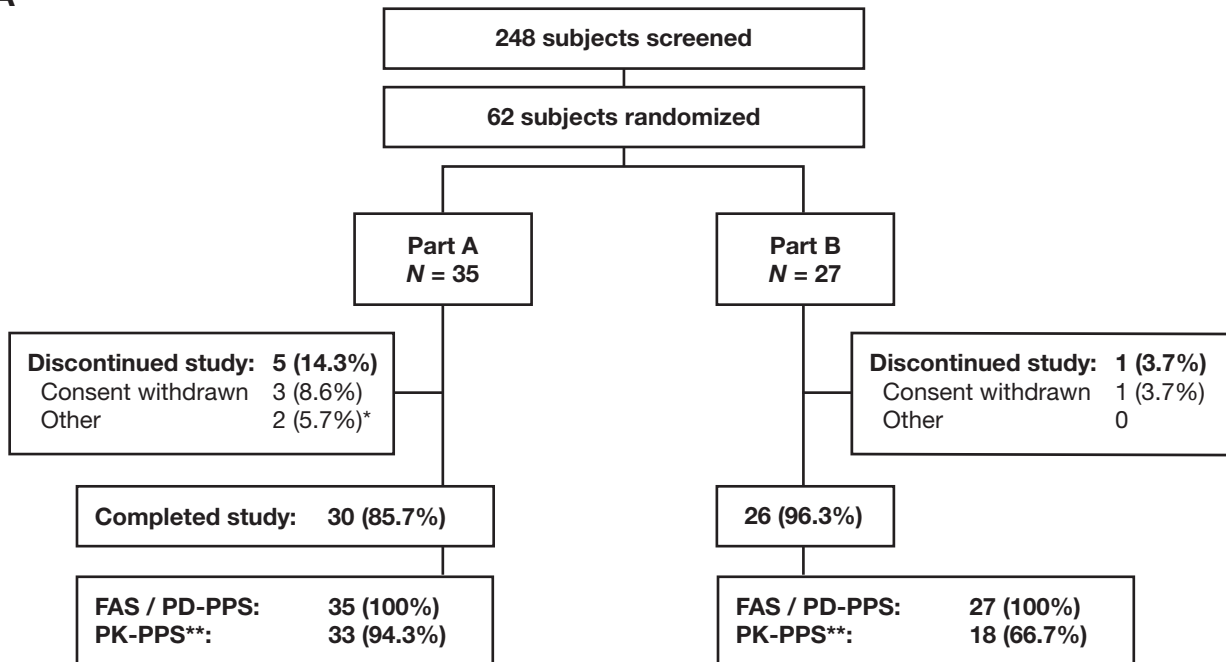

Supplement: Supplementary file 4 — Figure 2a (PDF 197 kb) [file 228_2017_2205_MOESM4_ESM.pdf]

**B**

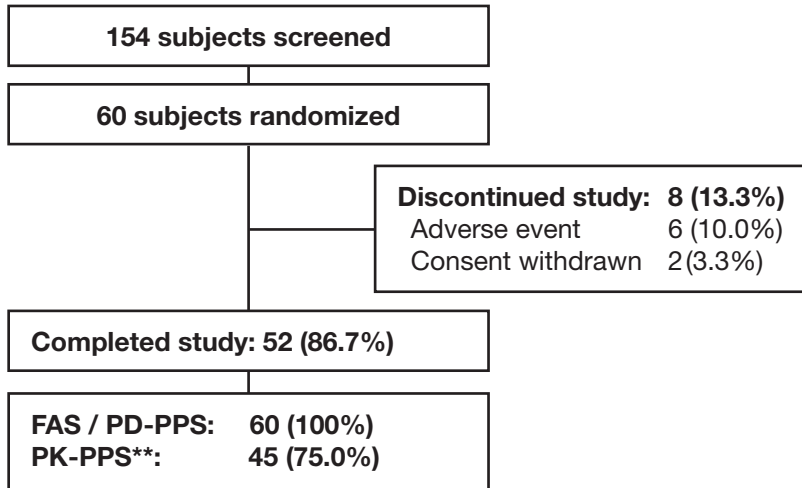

Supplement: Supplementary file 5 — Figure 2b (PDF 184 kb) [file 228_2017_2205_MOESM5_ESM.pdf]

**A**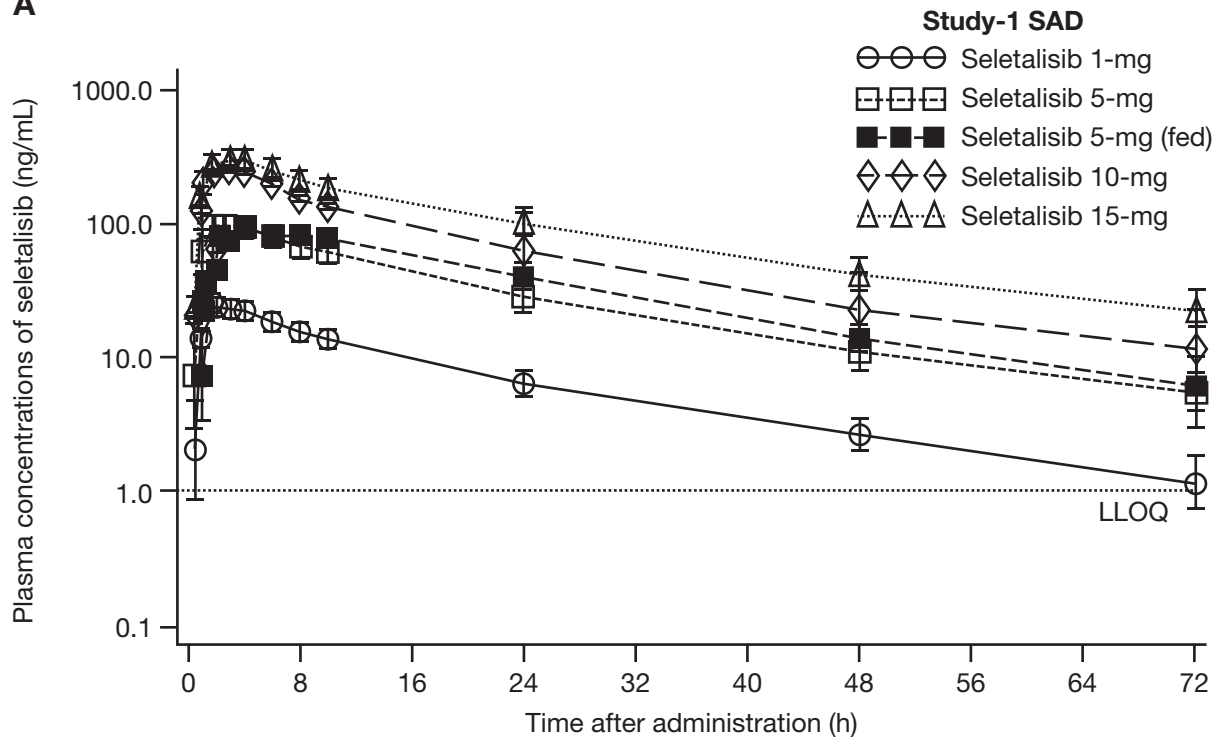

Supplement: Supplementary file 6 — Figure 3a (PDF 655 kb) [file 228_2017_2205_MOESM6_ESM.pdf]

**B**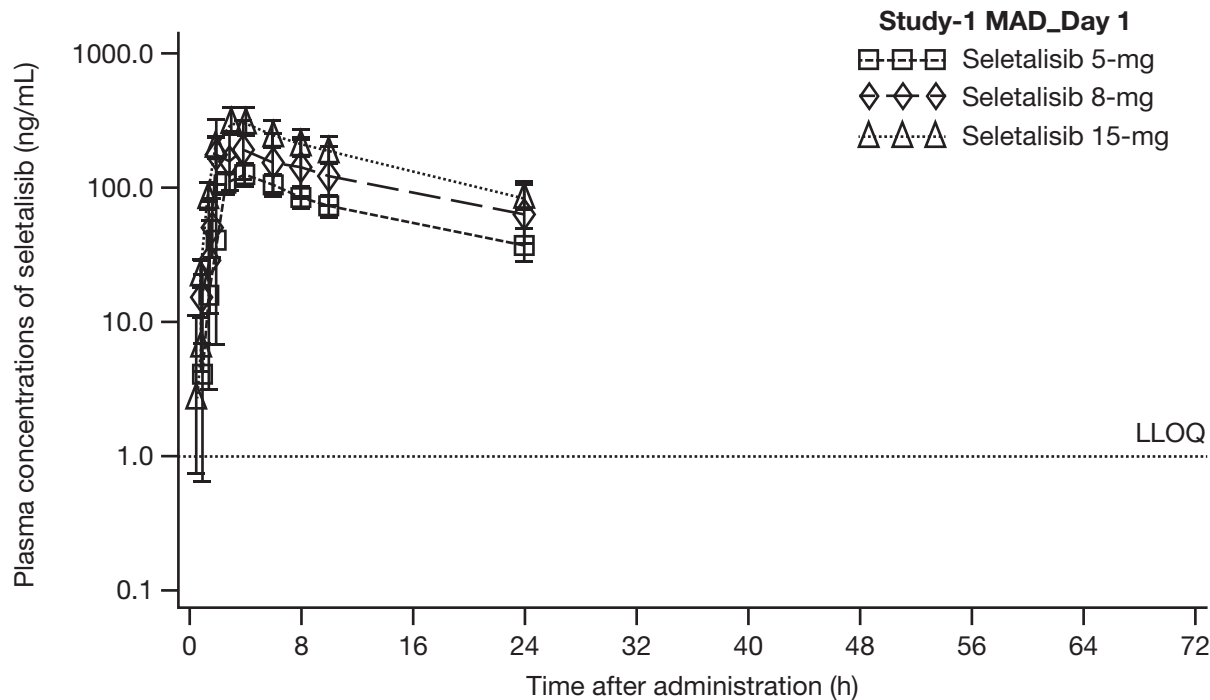

Supplement: Supplementary file 7 — Figure 3b (PDF 489 kb) [file 228_2017_2205_MOESM7_ESM.pdf]

**c**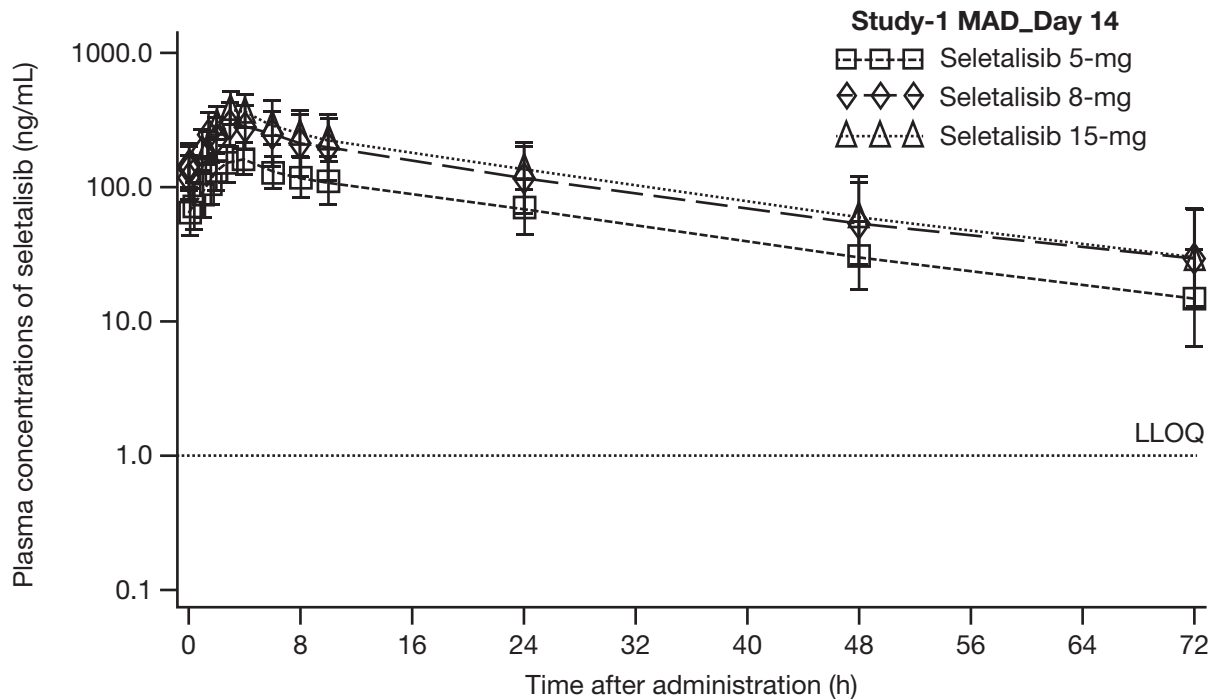

Supplement: Supplementary file 8 — Figure 3c (PDF 528 kb) [file 228_2017_2205_MOESM8_ESM.pdf]

**D**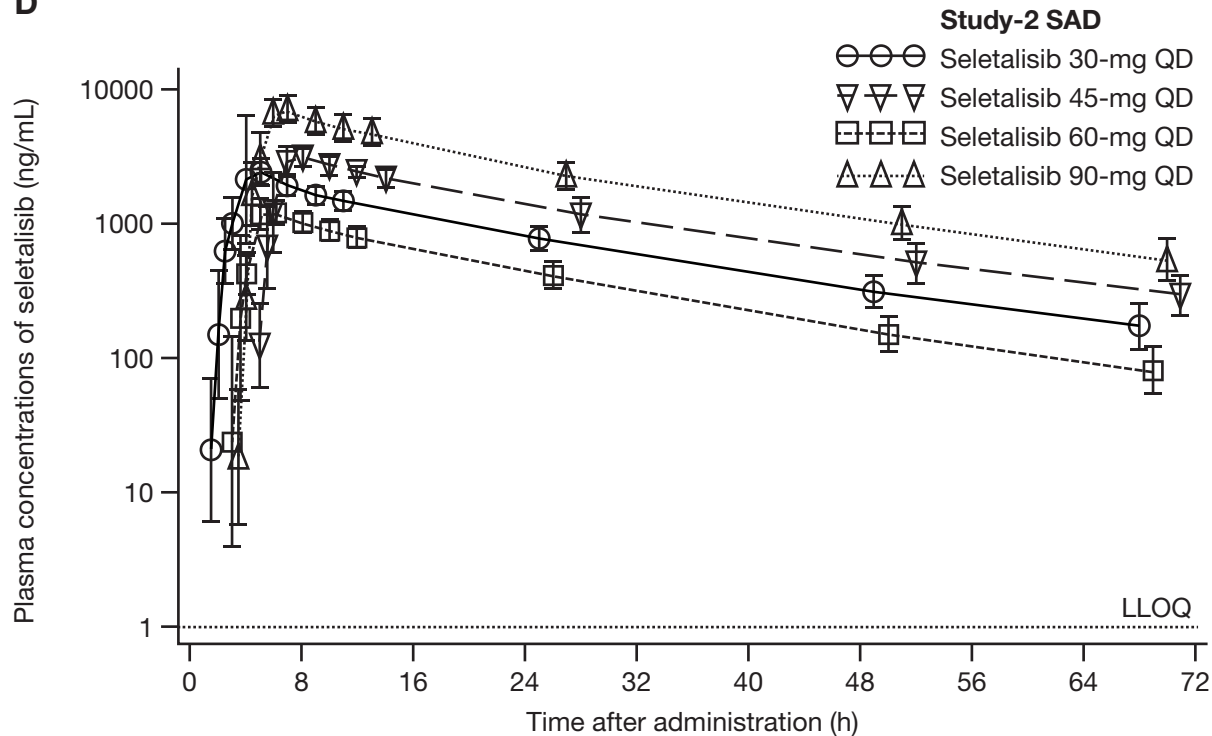

Supplement: Supplementary file 9 — Figure 3d (PDF 169 kb) [file 228_2017_2205_MOESM9_ESM.pdf]

**B****Study-1 MAD\_Day 1**

- Placebo
- Seletalisib 5-mg
- ◇—◇—◇ Seletalisib 8-mg
- △—△—△ Seletalisib 15-mg

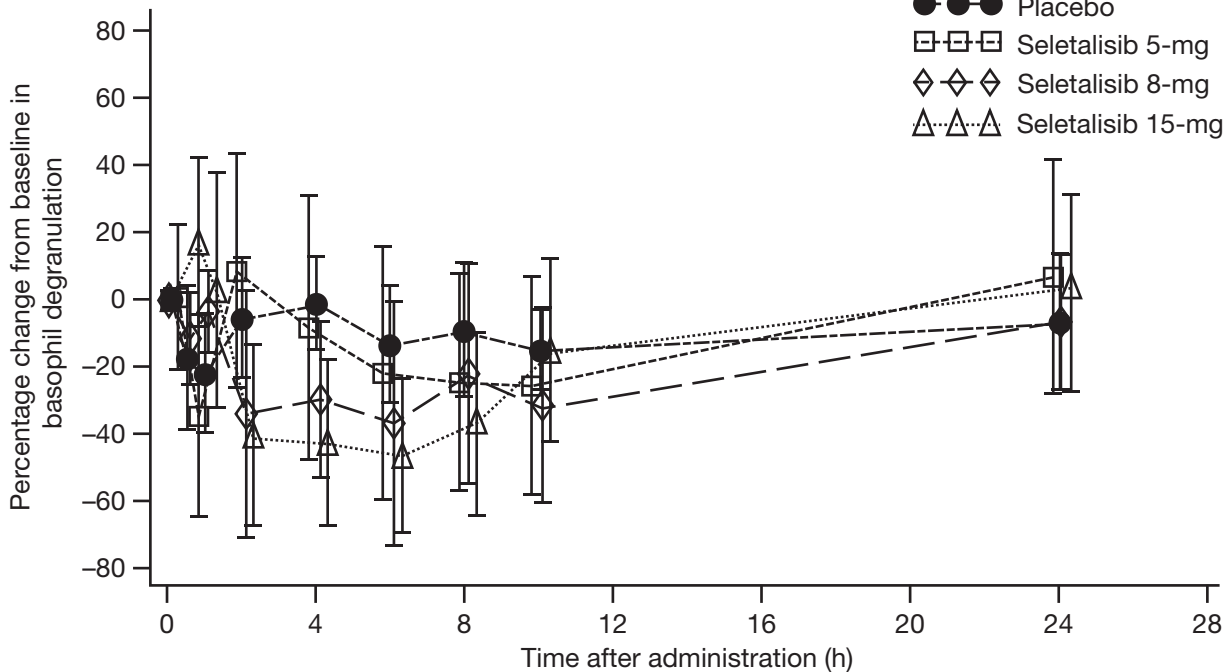

Supplement: Supplementary file 11 — Figure 4b (PDF 502 kb) [file 228_2017_2205_MOESM11_ESM.pdf]

**A**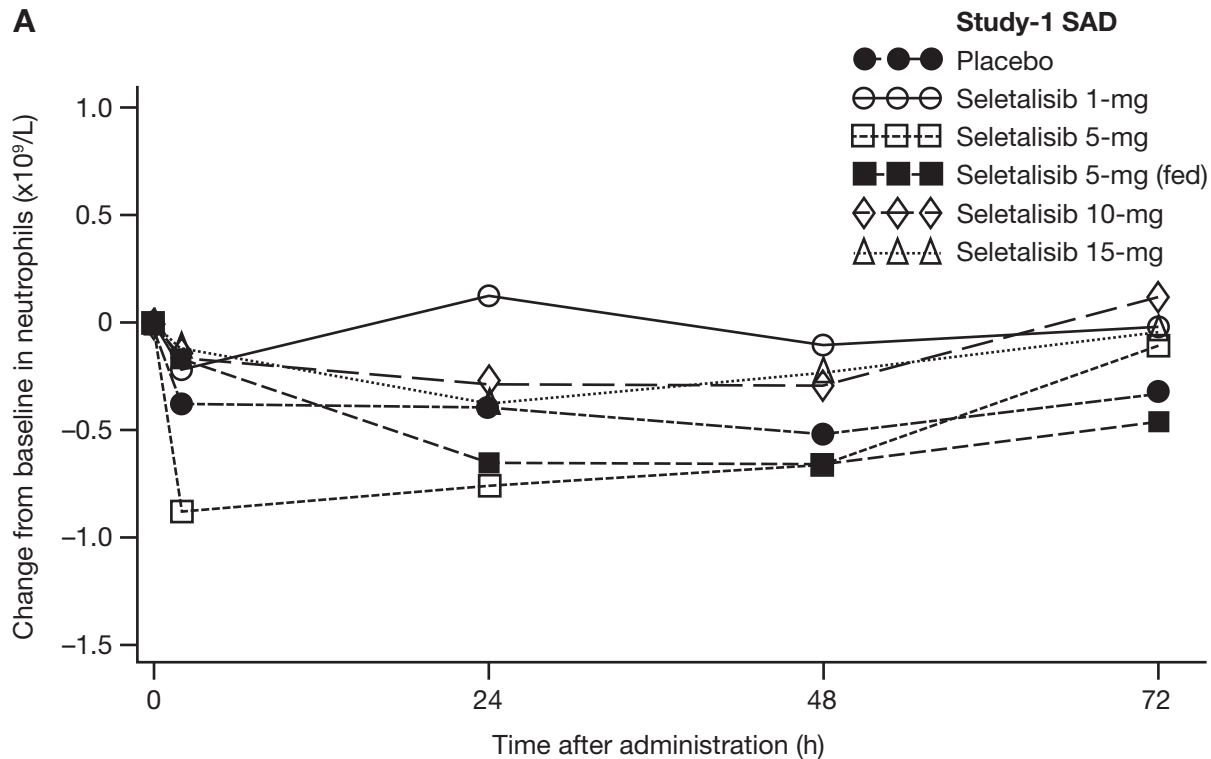

Supplement: Supplementary file 12 — Figure 5a (PDF 407 kb) [file 228_2017_2205_MOESM12_ESM.pdf]

# B

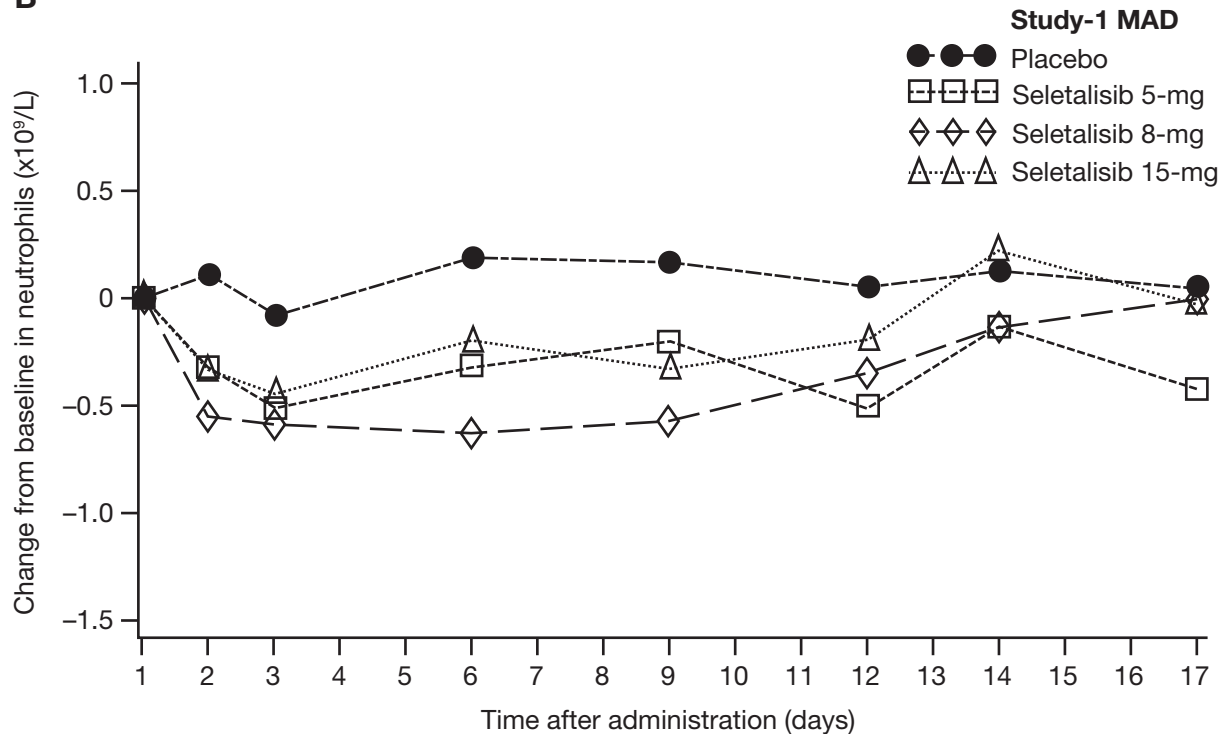

Supplement: Supplementary file 13 — Figure 5b (PDF 391 kb) [file 228_2017_2205_MOESM13_ESM.pdf]

Change from baseline in neutrophils ( $\times 10^9/L$ )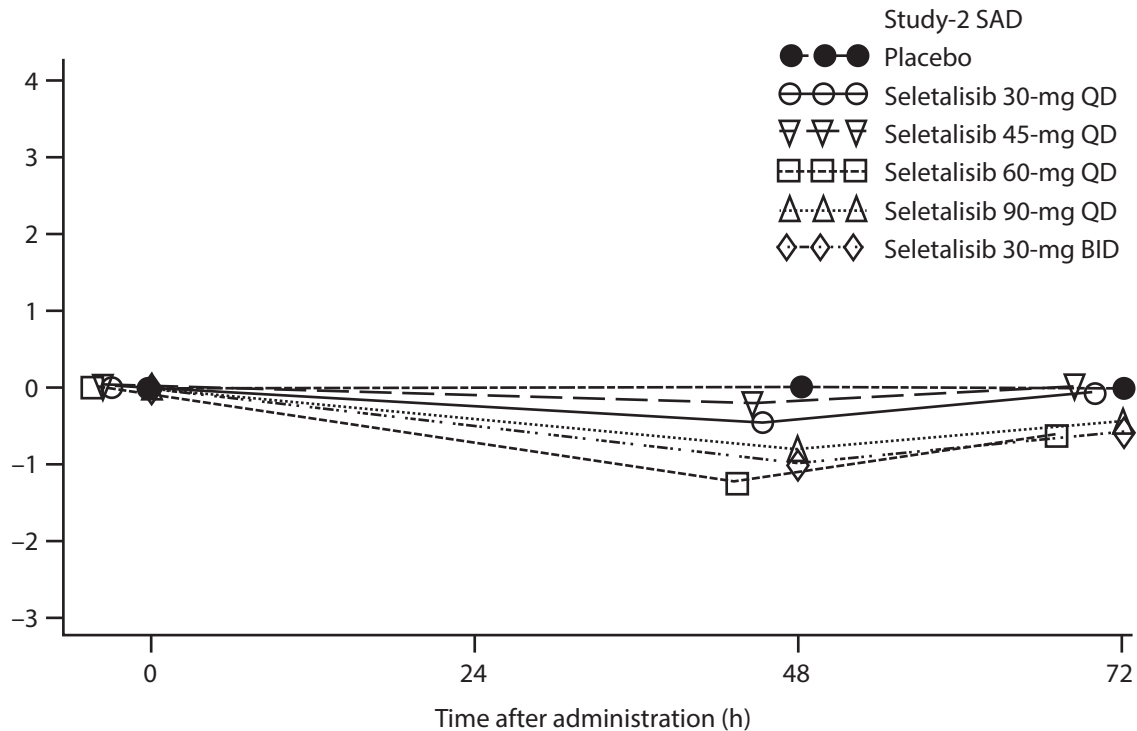

Supplement: Supplementary file 14 — Figure 5c (PDF 92 kb) [file 228_2017_2205_MOESM14_ESM.pdf]
