# Supplementary material for: First-in-human studies of seletalisib, an orally bioavailable small-molecule PI3Kδ inhibitor for the treatment of immune and inflammatory diseases
Source: Eur J Clin Pharmacol. 2017 Feb 4;73(5):581–91. doi: 10.1007/s00228-017-2205-7 (PMC5384962; doi:10.1007/s00228-017-2205-7)
Supplement: Supplementary file 10 — Figure 4a (PDF 570 kb) [file 228_2017_2205_MOESM10_ESM.pdf]

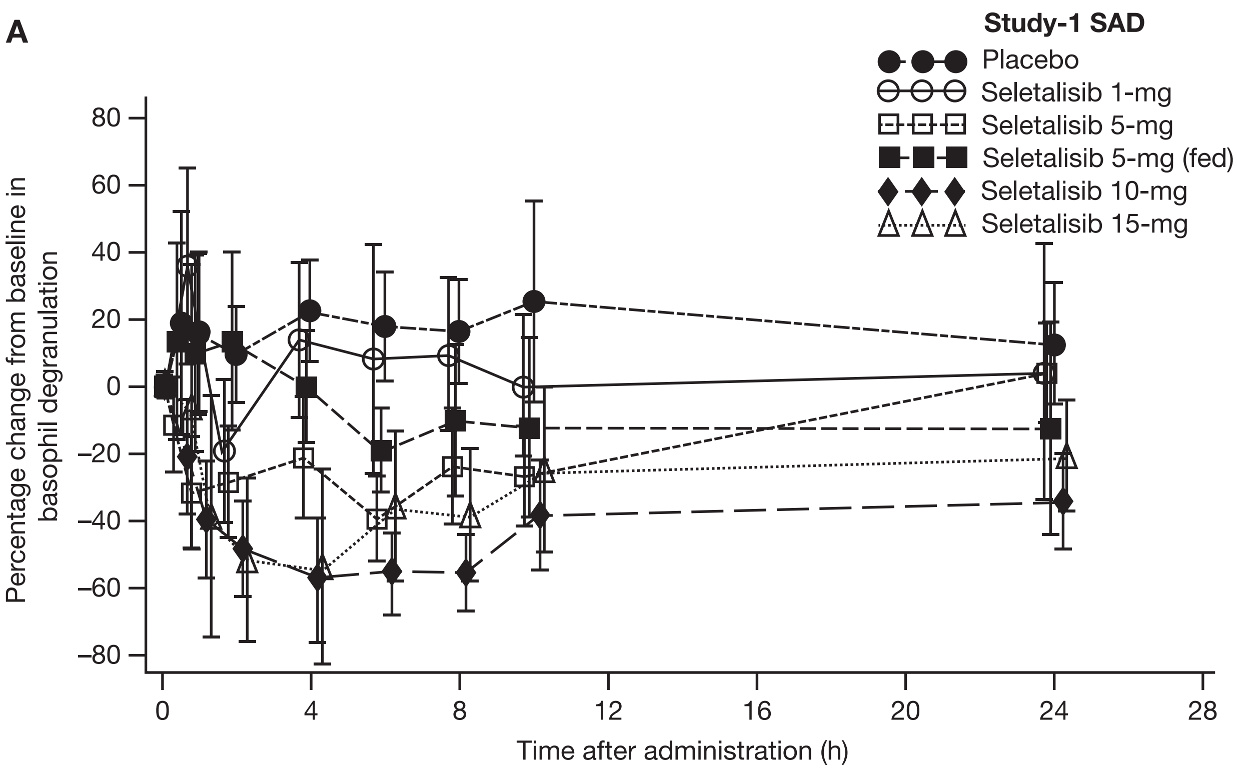

A
Study-1 SAD
Placebo
Seletalisib 1-mg
Seletalisib 5-mg
Seletalisib 5-mg (fed)
Seletalisib 10-mg
Seletalisib 15-mg
Percentage change from baseline in basophil degranulation
80
60
40
20
0
−20
−40
−60
−80
0
4
8
12
16
20
24
28
Time after administration (h)
